# An atlas of white matter anatomy, its variability, and reproducibility based on constrained spherical deconvolution of diffusion MRI

**DOI:** 10.1016/j.neuroimage.2022.119029

**Published:** 2022-02-26

**Authors:** Ahmed M. Radwan, Stefan Sunaert, Kurt Schilling, Maxime Descoteaux, Bennett A. Landman, Mathieu Vandenbulcke, Tom Theys, Patrick Dupont, Louise Emsell

**Affiliations:** a KU Leuven, Department of Imaging and pathology, Translational MRI, Leuven, Belgium; b KU Leuven, Leuven Brain Institute (LBI), Department of Neurosciences, Leuven, Belgium; c UZ Leuven, Department of Radiology, Leuven, Belgium; d Vanderbilt University Medical Center, Department of Radiology and Radiological Sciences, Nashville, TN, USA; e SCIL, Université de Sherbrooke, Quebec, Canada; f Vanderbilt University, Department of Electrical Engineering and Computer Engineering, Nashville, TN, USA; g KU Leuven, Department of Neurosciences, Neuropsychiatry, Leuven, Belgium; h KU Leuven, Department of Geriatric Psychiatry, University Psychiatric Center (UPC), Leuven, Belgium; i KU Leuven, Department of Neurosciences, Research Group Experimental Neurosurgery and Neuroanatomy, Leuven, Belgium; j UZ Leuven, Department of Neurosurgery, Leuven, Belgium; k KU Leuven, Laboratory for Cognitive Neurology, Department of Neurosciences, Leuven, Belgium

**Keywords:** Diffusion MRI, Tractography, Brain, White matter, CSD, Anatomy

## Abstract

Virtual dissection of white matter (WM) using diffusion MRI tractography is confounded by its poor reproducibility. Despite the increased adoption of advanced reconstruction models, early region-of-interest driven protocols based on diffusion tensor imaging (DTI) remain the dominant reference for virtual dissection protocols. Here we bridge this gap by providing a comprehensive description of typical WM anatomy reconstructed using a reproducible automated subject-specific parcellation-based approach based on probabilistic constrained-spherical deconvolution (CSD) tractography. We complement this with a WM template in MNI space comprising 68 bundles, including all associated anatomical tract selection labels and associated automated workflows. Additionally, we demonstrate bundle inter- and intra-subject variability using 40 (20 test-retest) datasets from the human connectome project (HCP) and 5 sessions with varying *b-*values and number of *b-*shells from the single-subject Multiple Acquisitions for Standardization of Structural Imaging Validation and Evaluation (MASSIVE) dataset. The most reliably reconstructed bundles were the whole pyramidal tracts, primary corticospinal tracts, whole superior longitudinal fasciculi, frontal, parietal and occipital segments of the corpus callosum and middle cerebellar peduncles. More variability was found in less dense bundles, e.g., the fornix, dentato-rubro-thalamic tract (DRTT), and premotor pyramidal tract. Using the DRTT as an example, we show that this variability can be reduced by using a higher number of seeding attempts. Overall inter-session similarity was high for HCP test-retest data (median weighted-dice = 0.963, stdev = 0.201 and IQR = 0.099). Compared to the HCP-template bundles there was a high level of agreement for the HCP test-retest data (median weighted-dice = 0.747, stdev = 0.220 and IQR = 0.277) and for the MASSIVE data (median weighted-dice = 0.767, stdev = 0.255 and IQR = 0.338). In summary, this WM atlas provides an overview of the capabilities and limitations of automated subject-specific probabilistic CSD tractography for mapping white matter fasciculi in healthy adults. It will be most useful in applications requiring a reproducible parcellation-based dissection protocol, and as an educational resource for applied neuroimaging and clinical professionals.

## 1. Introduction

Characterizing the macroscopic structural organization of brain connectivity in vivo is central to understanding the human nervous system in health and disease. The advent of diffusion magnetic resonance imaging (dMRI) fiber tractography (FT) more than twenty years ago enabled significant progress in mapping major white matter (WM) fiber bundles described in anatomical and surgical literature. Initial work based on Diffusion Tensor Imaging (DTI) (Basser et al., 1994a, 1994b; Mori et al., 1999) drove the development of white matter dissection protocols (Catani, 2006; Mori et al., 2009, 2008; Wakana et al., 2007), which are still widely used today. This is because DTI is a simple and effective approach for reconstructing the core of large fasciculi (Catani et al., 2002; Stieltjes et al., 2001), data acquisition for DTI modelling requires a relatively short scan time (5–10 min) and standard pulse sequences are widely available owing to their regulatory approval for clinical practice. However, it is widely accepted that DTI suffers limitations that make it suboptimal for many tractography applications, particularly in a clinical setting (Farquharson et al., 2013; Mori and Tournier, 2014; Tournier et al., 2011). This has led to increased interest in more accurate and reliable approaches using high angular resolution imaging (HARDI) data (Bayrak et al., 2020; Bloy et al., 2012; Wasserthal et al., 2018; Yeh et al., 2018). At the same time, technological advances to accelerate data acquisition and reconstruction, such as multiband (Bouyagoub et al., 2020; Duan et al., 2015; Larkman et al., 2001, p. 1; Moeller et al., 2010) and compressed sensing (Lustig et al., 2008, 2007) in combination with improved computational efficiency, will increase the adoption of advanced reconstruction models in applications traditionally reserved for DTI. This means that there is a need for updated reference material based on more representative virtual dissections using HARDI data acquired on 3T scanners.

Automated approaches relying on anatomical and orientational priors have been shown to considerably improve the accuracy of tract representations (Rheault et al., 2019) and address the poor reproducibility, and operator dependency that confound the manual virtual dissection process (Kreilkamp et al., 2019; Maffei et al., 2021; Soares et al., 2013).There are currently only a limited number of approaches that automate the virtual dissection process from start to finish (Warrington et al., 2020; Wasserthal et al., 2018; Yendiki et al., 2011). However, often the underlying dissection protocol is not explicitly detailed or the workflow is based on model bundles defined a priori.

This work addresses both the currently unmet need for an updated HARDI human white matter atlas that is relevant for clinical research studies, and a standardized, reproducible virtual dissection approach based on anatomical definitions. Using CSD due to its versatility and potential application to clinical data (Calamuneri et al., 2018; Toselli et al., 2017; Wilkins et al., 2015) we extend earlier virtual dissection protocols based on DTI by providing a descriptive summary of the normal WM anatomy of 68 fiber bundles reconstructed using probabilistic tractography. We complement our anatomical descriptions with an open-source group atlas in MNI space and automated subject-specific virtual dissection software, which we call “Fun with Tracts” (FWT) that incorporates all the anatomical inclusion and exclusion labels per bundle. As probabilistic tractography produces inherently variable results, we also demonstrate how this may vary within and between individuals using open-source test-retest datasets from the human connectome project (HCP) (Van Essen et al., 2012) and the Multiple acquisitions for standardization of structural imaging validation and evaluation (MASSIVE) dataset (Froeling et al., 2017). The result is a detailed and accessible reference for the virtual dissection of normal white matter anatomy using CSD tractography.

## 2. Material and methods

### 2.1. Summary

First, we describe and present an atlas of bundles generated with our literature-based dissection protocol (FWT) for 68 WM fasciculi based on the HCP-template data using a bundle-specific seeding and tractography approach described in more detail below. Tractogram images were generated using MRView (Tournier et al., 2019) and Surf-Ice (Rorden and Hanayik, 2022).

Second, we assessed inter-session/intra-subject variability by applying the FWT whole-brain tractography and bundle segmentation approach to the individual HCP test-retest datasets (Van Essen et al., 2012) and conducted similarity analyses between the resulting tractograms compared to the HCP-template bundles. Resulting weighted-dice similarity coefficient (wDSC) scores are shown in radar plots and were used to calculate single-rater agreement intra-class correlation (ICC) scores to highlight tractogram variability with respect to the template bundles. Additionally, we calculated descriptive statistics and generated violin plots for the wDSC scores resulting from pairwise comparisons of the output from each session (i.e., HCP scan 1 versus HCP scan 2). This provided a measure of FWT reproducibility within and between subjects regardless of similarity to a model bundle. HCP inter-subject variability was evaluated by generating voxel-wise heat-maps of the summed binary masks of each bundle.

Third, to test the variability of FWT in an independent dataset with different acquisition parameters and sampling schemes, we applied it to the MASSIVE dataset (Froeling et al., 2017). Model-based wDSC bundle similarity scores, using the HCP-template bundles as references, were then calculated for every bundle from both the HCP and MASSIVE datasets. We computed wDSC descriptive statistics and generated violin plots for evaluation. This provided a framework for the comparison of the outputs from both datasets. Finally, we used single-rater agreement ICC scores to evaluate overall inter-session agreement.

### 2.2. Imaging data

#### 2.2.1. HCP test‐retest data

The test-retest HCP dataset (Van Essen et al., 2012) (https://db.humanconnectome.org/data/projects/HCP_Retest) consists of 2 scans acquired at 1 – 11 months apart using the same scanning protocol on the same 3-Tesla Siemens Skyra scanner (Siemens Healthineers, Erlangen, Germany) using a 32-channel phased array receive head coil. The HCP T1-weighted images were acquired using a 3D Magnetization Prepared Rapid Acquisition Gradient Echo (MPRAGE) pulse sequence with 0.7 mm isotropic voxels. The diffusion data was acquired with 90 directions per shell with *b-*values (1000, 2000, and 3000 s/mm^2^), and 1.25 mm isotropic voxels. We used the preprocessed (Andersson et al., 2003a; Andersson and Sotiropoulos, 2016, 2015; Glasser et al., 2013; Jenkinson et al., 2002) imaging data of 20 random subjects (10 male / 10 female) resulting in 40 scans (see supplementary Table 1 for details).

#### 2.2.2. MASSIVE data

The MASSIVE dataset (Froeling et al., 2017) (http://www.massive-data.org), comprises multiple scans of the same healthy individual (female, 25 years old) using various *b-*values and diffusion sampling schemes. Imaging data was acquired on a 3-Tesla Philips Achieva scanner (Philips Healthcare, Best, The Netherlands) with an 8-channel phased array receive head coil. MASSIVE 3D T1-weighted images were acquired with 1 mm isotropic voxels, while dMRI images were acquired with 2.5 mm isotropic voxels and multiple shells with (0–9000 s/mm^2^) *b-*values. We used the (0–4000 s/mm^2^) *b-*shell data with the following *b-*values in s/mm^2^ and number of diffusion-weighting gradient directions respectively (b500 – 125, b1000 – 250, b2000 – 250, b3000 – 250, b4000 – 300). Five different sessions were generated using the preprocessed dMRI data acquired with an anterior-posterior (AP) phase-encoding axis and a negative gradient polarity to create 5 different sessions. Two sessions had multi-shell and three had single shell data with varying *b-*values, numbers of diffusion-weighted volumes and interleaved b0 volumes. Additionally, we used corresponding reversed-phase encoded b0 images for Echo-Planar Imaging (EPI) distortion correction in FSL (Jenkinson et al., 2012). We used the MASSIVE data to investigate FWT reproducibility in the same subject with different *b-*values, number of diffusion directions and *b-*shells Table 1. lists the *b-*values and total number of volumes of all dMRI data used.

### 2.3. Imaging data analysis

#### 2.3.1. Image preprocessing

All data was arranged in a Brain Imaging Data Structure (Gorgolewski et al., 2016) (BIDS) convention so that MultiScale Brain Parcellator (MSBP) (Tourbier et al., 2019) and FWT could run automatically. The T1-weighted images from both the HCP test-retest and the MASSIVE datasets were parcellated using FreeSurfer (Fischl, 2012) and MSBP (Tourbier et al., 2019). We used MRTrix3 (Tournier et al., 2019) and FSL (Jenkinson et al., 2012) for dMRI preprocessing. The HCP test-retest data did not undergo any additional preprocessing. The MASSIVE diffusion data were preprocessed to remove any residual noise (Cordero-Grande et al., 2019; Veraart et al., 2016), motion/eddy and EPI related artefacts and distortions (Andersson et al., 2003a, 2003b; Andersson and Sotiropoulos, 2016; Bastiani et al., 2019; Skare and Bammer, 2010; Smith et al., 2004). fODF maps were generated using Multi-shell Multi-tissue CSD (Jeurissen et al., 2014) (MSMT-CSD) for all data, with three tissue types for multi-shell data and two tissue types of single-shell data.

#### 2.3.2. HCP test‐retest group template creation

We created group averaged T1-weighted images and WM fODF maps from each individual HCP dataset in MRTrix3 (Tournier et al., 2019), referred to throughout this work as the HCP-template for simplicity. The resulting averaged T1-weighted image was parcellated using FreeSurfer (Fischl, 2012) and MSBP (Tourbier et al., 2019).

#### 2.3.3. Virtual white matter dissection protocol

We reconstructed the following 68 WM fiber bundles using FWT based on anatomical and neuroimaging literature definitions, 15 bilateral association bundles: The arcuate fasciculus, cingulum, fornix, frontal aslant tract, Inferior fronto-occipital fasciculus, inferior and middle longitudinal fasciculi, whole superior longitudinal fasciculus and its subcomponents, uncinate fasciculus, vertical occipital fasciculus. 8 commissural bundles: the anterior commissure, and corpus callosum in 7 segments. 12 bilateral projection bundles: the medial lemniscus, optic pathway, whole pyramidal tract and its subcomponents, and thalamic radiations. 3 bilateral cerebellar bundles: Dentato-rubro-thalamic tract, inferior and middle cerebellar peduncles. Definitions for each bundle are described in the results section and the inclusion/exclusion VOIs are provided in the supplementary methods.

#### 2.3.4. FWT

**Fig. 1.**
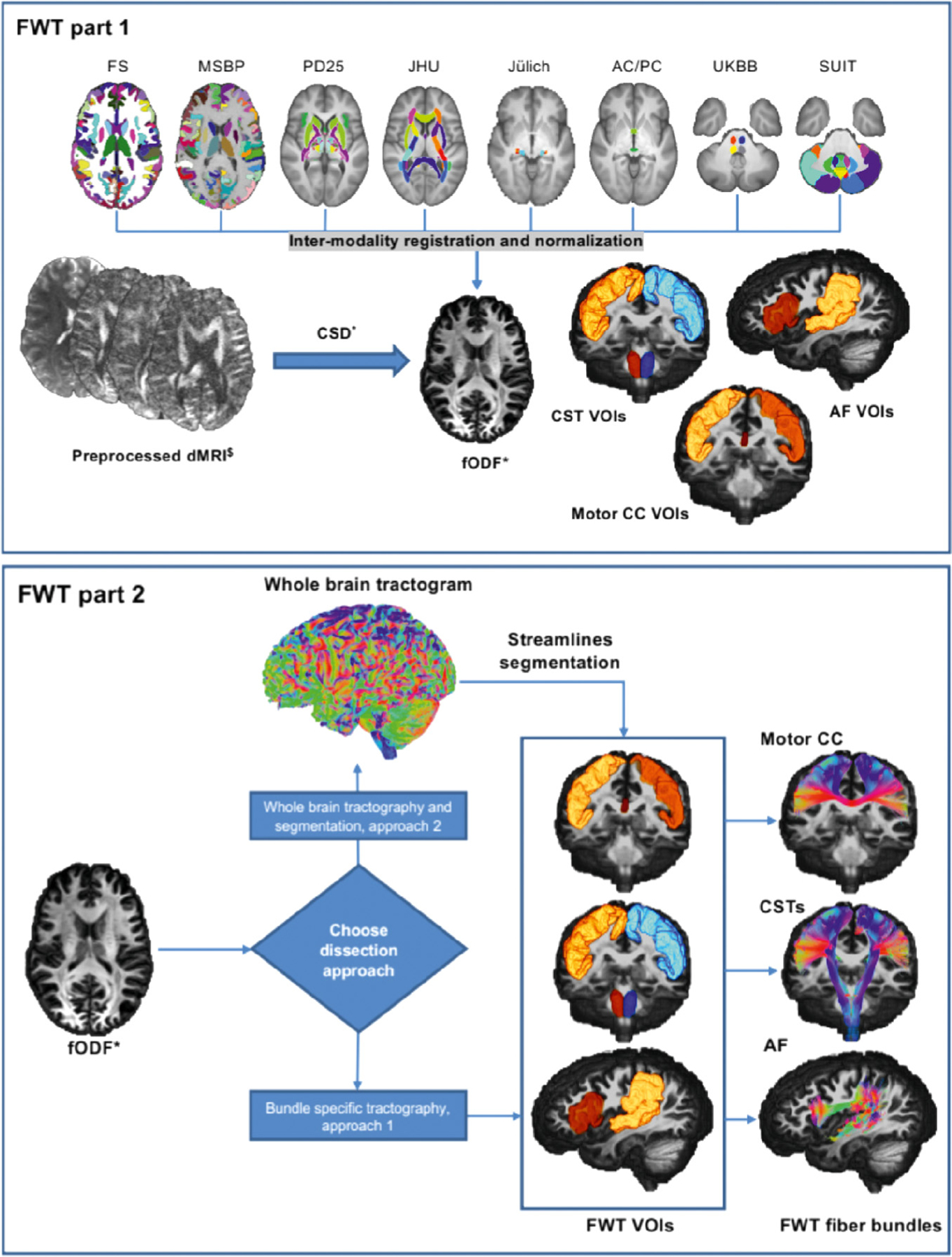
Graphical representation of the 2 parts of FWT for the corticospinal tracts (CSTs), arcuate fasciculi (AF), and motor corpus callosum (CC). FWT part 1 (top) requires as input preprocessed diffusion data, and FS recon-all output, and MSBP output. The script generates all VOIs used for virtual dissection by combining various anatomical VOIs from different parcellation maps and atlases. FWT part 2 (bottom) generates all tractograms from preprocessed diffusion data and VOIs created by FWT part 1, this script provides two approaches to virtual dissection; (1) Bundle specific and (2) Whole brain tractography followed by segmentation. FS = FreeSurfer, MSBP = MultiScale Brain Parcellator, PD25 = NIST Parkinson’s histological, JHU = John’s Hopkins university, Juelich = Juelich university histological atlas, AC/PC = anterior commissure/posterior commissure, manually defined VOIs in template space, UKBB = UK Biobank, SUIT = spatially unbiased cerebellar atlas template, dMRI = diffusion magnetic resonance imaging, CSD = constrained spherical deconvolution, fODF = fiber orientation distribution function. $ = preprocessed, should include correction for motion, Eddy currents, EPI distortion, imaging noise and bias. ∗ = Other models e.g., DTI with FACT.

Fig. 1 provides a schematic description of FWT. A detailed technical description including tracking and optimization parameters is provided in supplementary methods.

The automated workflows can be found at (https://github.com/KUL-Radneuron/KUL_FWT.git). FWT employs automated tractography using MRTrix3 (Tournier et al., 2019) v3.0.2, which is constrained by a selection of grey matter and WM 3D VOIs/parcels used to create “inclusion” and “exclusion” areas, based on the neuroanatomical literature. These VOIs/parcels are obtained from FreeSurfer (Fischl, 2012) v6 (FS), MSBP (Tourbier et al., 2019) v1.1.1, and several a priori atlases (see supplementary materials), along with custom VOIs manually defined in template space (the anterior commissure midline, and posterior commissure VOIs), and other custom VOIs generated by label propagation, (e.g., su*b-*segmentation of the periventricular white matter, temporal stem, insula, and superior temporal gyrus subcortical white matter). Streamlines filtering is done using ScilPy (Bore et al., 2021; “Scilpy documentation,” 2021) v.1.1.0 and DIPY (Garyfallidis et al., 2014) tools and v1.3.0 for all bundles except the optic radiations for which we used the fiber-to-Bundle coherence (FBC) (Meesters et al., 2017; Portegies et al., 2015) tool in DIPY (Garyfallidis et al., 2014) v1.3.0. FWT can be used for individual bundle tractography or whole brain tractography and streamlines segmentation.

#### 2.3.5. Creation of inclusion and exclusion VOIs

The first part of the FWT workflow automatically generates the inclusion and exclusion VOIs using the outputs of FS (Fischl, 2012) recon-all (FreeSurferWiki, 2020), MSBP (Tourbier et al., 2019), along with the UK BioBank (UKBB) volumetric atlas of fiber bundles (Miller et al., 2016), the spatially unbiased atlas template of the cerebellum and brainstem (Diedrichsen, 2006; Diedrichsen et al., 2011, 2009; Diedrichsen and Zotow, 2015) (SUIT), the Neuroimaging and surgical technologies (NIST) Parkinson’s disease histological atlas (Xiao et al., 2017, 2015, 2012) (PD25), and manually defined VOIs for the anterior and posterior commissures. The resulting anatomical VOIs are warped to diffusion space using ANTs (Avants et al., 2011), then combined to form bundle specific inclusion and exclusion VOIs. In our analysis, these VOIs were applied to each individual dataset (i.e., the two sessions of each subject in the HCP test-retest dataset, and the different scan sessions of the preprocessed MASSIVE single subject dataset). A similar workflow designed for group template T1 images was used on the HCP-template data.

#### 2.3.6. Individual tractography and creation of FWT‐HCP template bundles

The second part of the FWT workflow provides the choice of two different whole brain WM virtual dissection approaches, both of which rely on the inclusion and exclusion VOIs created by the first part of FWT. The first approach employs bundle-specific seeding followed by streamline filtering and smoothing. The second generates a whole brain tractogram with 10 million streamlines by default followed by streamline dissection, then streamline filtering and smoothing. An equivalent workflow for fODF group averaged maps is also available generating whole brain tractograms with 20 million streamlines by default.

For this work we relied on bundle specific tractography to generate the 68 HCP-template bundles, and whole brain tractography followed by bundle dissection for all individual datasets. All template tractograms underwent visual quality assurance prior to further use. A tractogram is considered to have failed if less than 10 streamlines are generated initially, or if less than 10 streamlines are found after the first filtering step. We only included the successfully generated final FWT output tractograms in the current analysis.

#### 2.3.7. Tractogram reproducibility measures

Tractograms can be used to generate segmentation maps that can be compared using well-known similarity/dissimilarity measures such as Hausdorff distance, overlap measures, DSC, etc. As streamline specific variants of these similarity measures have been shown to underestimate tractogram similarity (Rheault et al., 2020) we used voxel-based weighted-dice similarity (wDSC) scores to simultaneously assess overlap and streamline density agreement per voxel in our inter-subject and intra-subject variability analyses (Cousineau et al., 2017). Additional similarity measures and their definitions, e.g., DSC, density correlation (Rheault et al., 2020), volume overlap and overreach (Maier-Hein et al., 2017), and bundle adjacency (Garyfallidis et al., 2012) are provided in the supplementary material along with voxel-wise cumulative maps for each bundle to demonstrate inter-subject variability.

## 3. Results

### 3.1. Qualitative bundle descriptions, template output and sample variability

Detailed qualitative descriptions of the virtual dissection protocol and demonstrative figures of bundles reconstructed from the HCP-template are provided below. Bundles are grouped by type (Meynert, 1888), i.e., association, commissural, and projection bundles, and cerebellar bundles are grouped separately. Radar-plots show the wDSC scores for each bundle, and wDSC summary statistics for each bundle group are included in supplementary table 3. Overall single-score agreement ICC was 0.713 (upper-bound = 0.789, lower-bound = 0.637, *P* < 0.05).

#### 3.1.1. Association fiber bundles

##### 3.1.1.1. Arcuate fasciculus (AF).

**Fig. 2.**
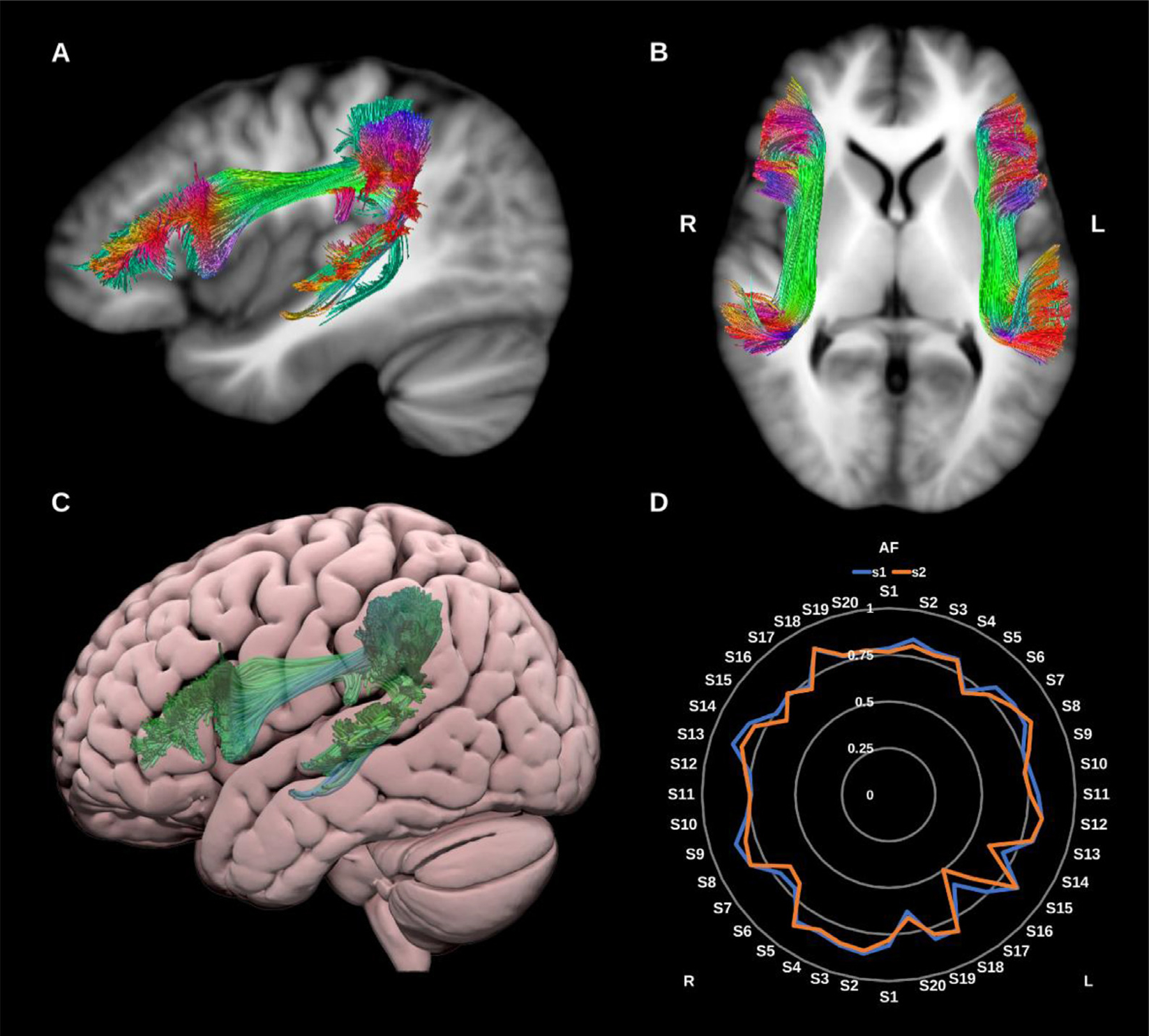
(A) and (B) Arcuate fasciculi (AF) overlaid in directional color coding on T1-weighted images. (C) 3D lateral projection of the left arcuate fasciculus in green overlaid on semitransparent MNI pial surface. (D) Radar plot of the wDSC scores (vertical range) of both AFs using first session (blue) and second session (orange). L = left, R = right, MNI = Montreal Neurological Institute, S = subject, wDSC = weighted dice similarity coefficient.

The AF is a major component of the dorsal language stream. It constitutes a perisylvian fronto-temporal pathway consisting of a long ‘direct’ association fiber system and two shorter ‘indirect’ bundles, see Fig. 2.

The direct bundle connects the ventral precentral, posterior inferior and middle frontal gyri with the middle and superior temporal gyri, and the two shorter bundles: (1) an anterior network connecting the supra- marginal and superior temporal gyri with the precentral gyrus, and (2) a posterior network connecting the posterior middle temporal gyrus with the angular gyrus (Bain et al., 2019; Bernard et al., 2019; Catani, 2006; Chen et al., 2015; Fernández-Miranda et al., 2015; Wakana et al., 2007; Wang et al., 2016; Yeh et al., 2018).

##### 3.1.1.2. Cingulum (CG/Cing).

**Fig. 3.**
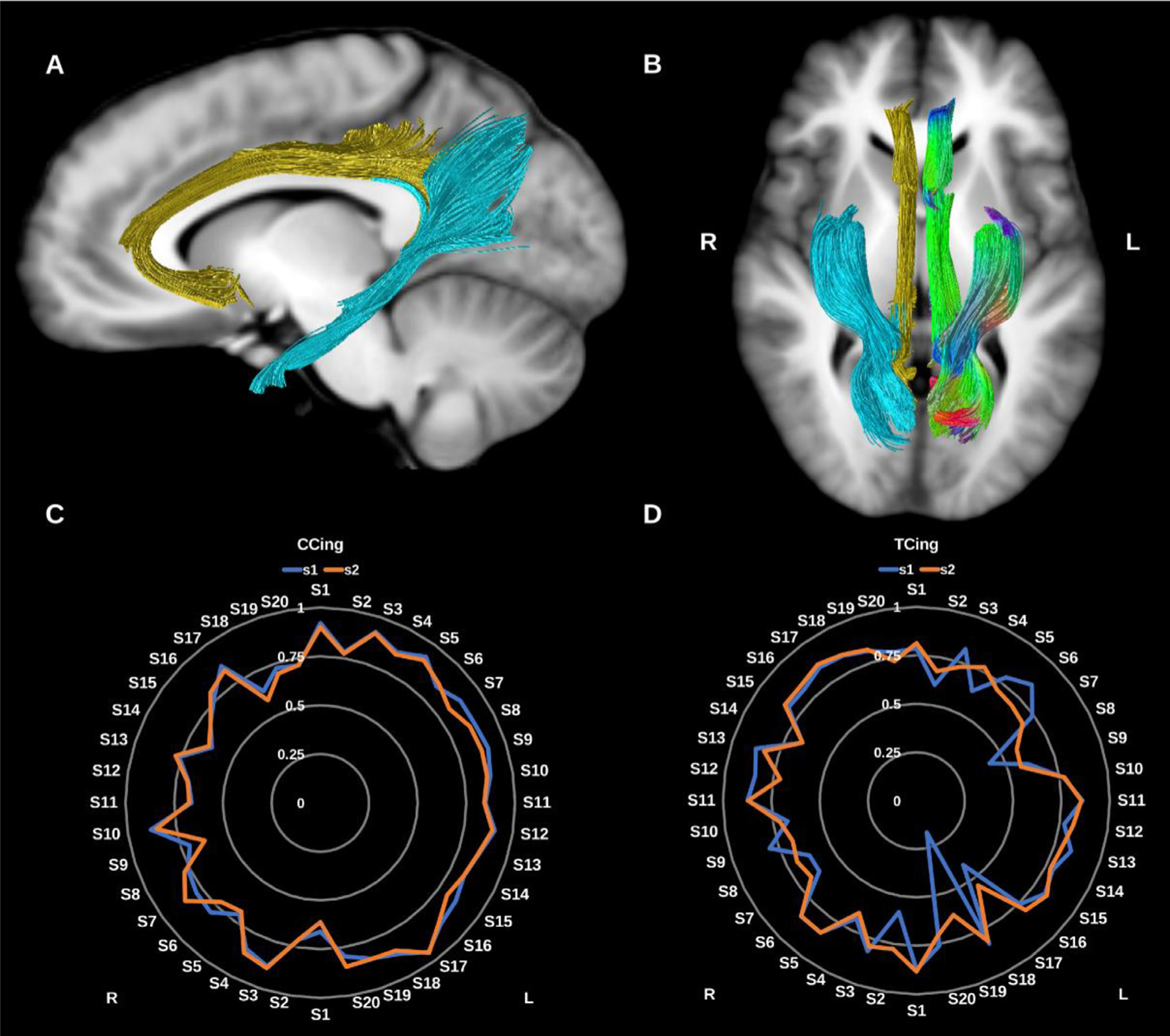
(A) and (B) Cingulum bundles overlaid in directional color coding for the left side and in gold for the cingulate portion (CCing) and light blue for the temporal portion (TCing) on sagittal and axial T1-weighted images. (C) and (D) Radar plots of the wDSC scores (vertical ranges) of the CCings and TCings reconstructed using first session (blue) and second session (orange). L = left, R = right, S = subject, wDSC = weighted dice similarity coefficient.

The cingulum bundle (CG) is the principal WM tract of the cingulate gyrus and the limbic system and is involved in a diverse range of functions spanning emotional, behavioral and sensorimotor control, mnemonic processing, nociception and executive function. Broadly speaking, the CG is a bidirectional fiber system that encircles the corpus callosum lateral to the cingulate gyrus, extending from the frontal lobes to the WM of the ventral temporal lobe with numerous lateral projections joining and leaving the bundle along its path (Jones et al., 2013; Metzler-Baddeley et al., 2012; Pascalau et al., 2018; Wu et al., 2016b), see Fig. 3.

The complexity of CG connections is inadequately characterized by dMRI, which tends to reconstruct either a single unilateral bundle extending from the subgenual cingulate to the temporal lobe, or two subdivisions encompassing the subgenual-dorsal, and dorsal-temporal segments. However, primate tracer studies suggest the CG could be subdivided into three or four regions based on differences in anatomical connectivity and neurotransmitter profile (Heilbronner and Haber, 2014). Here we reconstruct two su*b-*divisions in keeping with previous dMRI literature.

###### 3.1.1.2.1. Cingulate Cingulum (CCing).

This represents the dorsal (cingulate) component of the CG and is reconstructed by tracking the streamlines between the rostral and caudal anterior cingulate cortex and the posterior cingulate, isthmic posterior cingulate cortex and the precuneus. It is immediately superior to the body of the corpus callosum. It has a low curvature C shape, tipped on its open end with anterior/ventral extension to the subgenual cortex and ends posteriorly behind the splenium of the corpus callosum.

###### 3.1.1.2.2. Temporal Cingulum (TCing).

This represents the ventral component of the CG and is reconstructed by tracking the streamlines between the hippocampus and the isthmic posterior cingulate cortex and precuneus posteriorly. It is located in the medial temporal lobe, ascending from the anterior medial temporal lobe to the midline parietal region, behind the splenium of the corpus callosum.

##### 3.1.1.3. Fornix (FX).

**Fig. 4.**
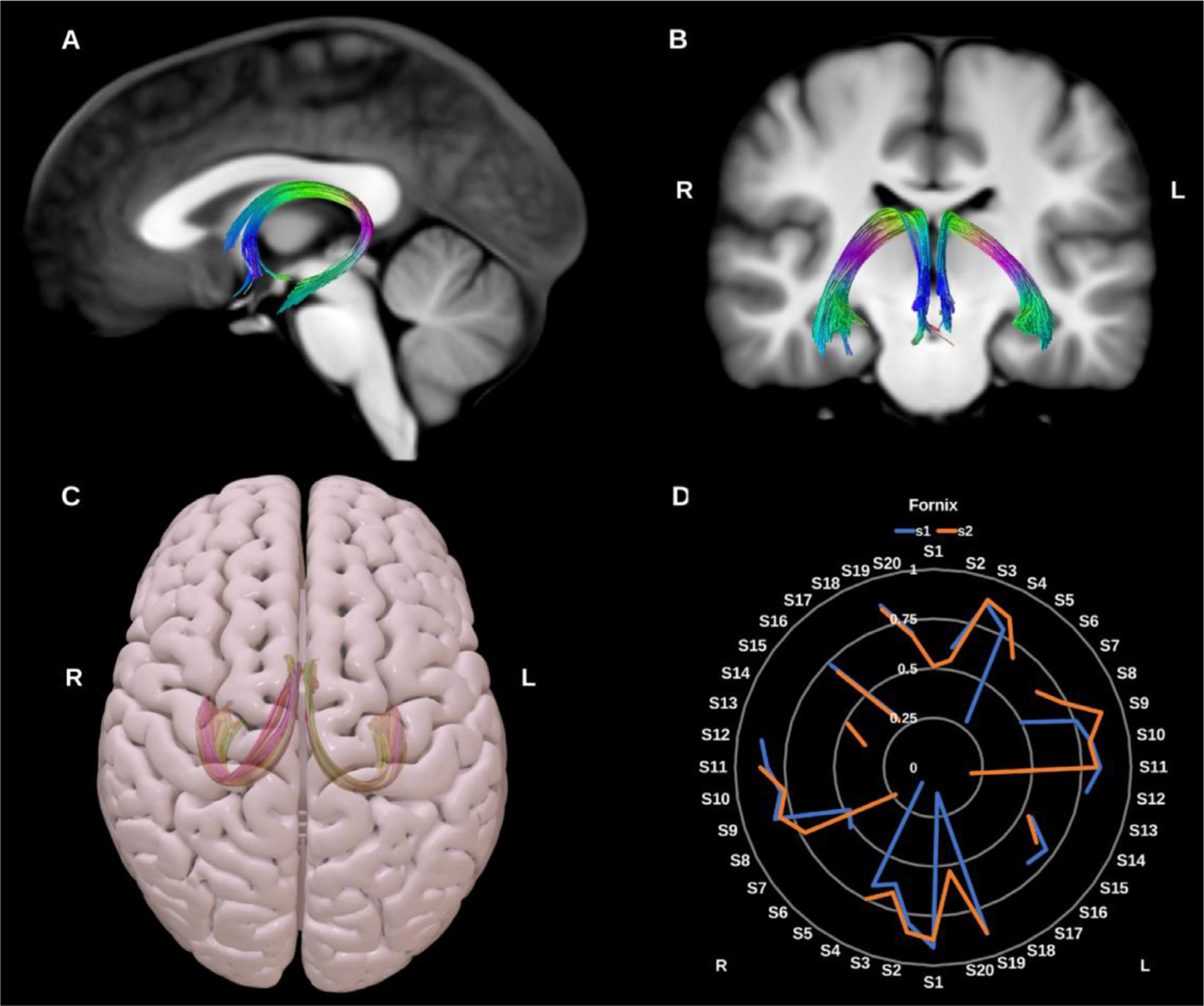
(A) and (B) Fornix (Fx) overlaid in directional color coding on sagittal and coronal slices of T1-weighted images. (C) 3D superior projection of the semitransparent MNI pial surface with both fornices shown in yellow and red. (D) Radar plot of the wDSC scores (vertical range) of both fornices reconstructed using first session (blue) and second session (orange). A missing line indicates a missing bundle for that dataset. L = left, R = right, S = subject, MNI = Montreal Neurological Institute, wDSC = weighted dice similarity coefficient.

The fornix is the major fiber pathway associated with the hippocampus, and comprises predominantly efferent fibers connecting the hippocampus with the prefrontal cortex, the anterior thalamic nuclei, the mammillary bodies, the ventral striatum, and the basal forebrain. Initially formed by the alveus and fimbria, WM of the bilateral hippocampus coalesce as the fornix crus and body before diverging again into the pre-commissural and post-commissural fornix columns which derive their name from their position relative to the anterior commissure. Recent research suggests fibers within the fornix are arranged topographically reflecting functional anterior-posterior gradient along the long axis of the hippocampus, with laterally located fibers arising from the anterior hippocampus and medially located fibers originating in the posterior hippocampus (Christiansen et al., 2017; Liacu et al., 2012; Pascalau et al., 2018; Strange et al., 2014), see Fig. 4. Here we reconstruct the fornix as a single lateralized bundle for each side. Reproducibility was poor and reconstruction failed in 19 datasets.

##### 3.1.1.4. Frontal aslant tract (FAT)

**Fig. 5.**
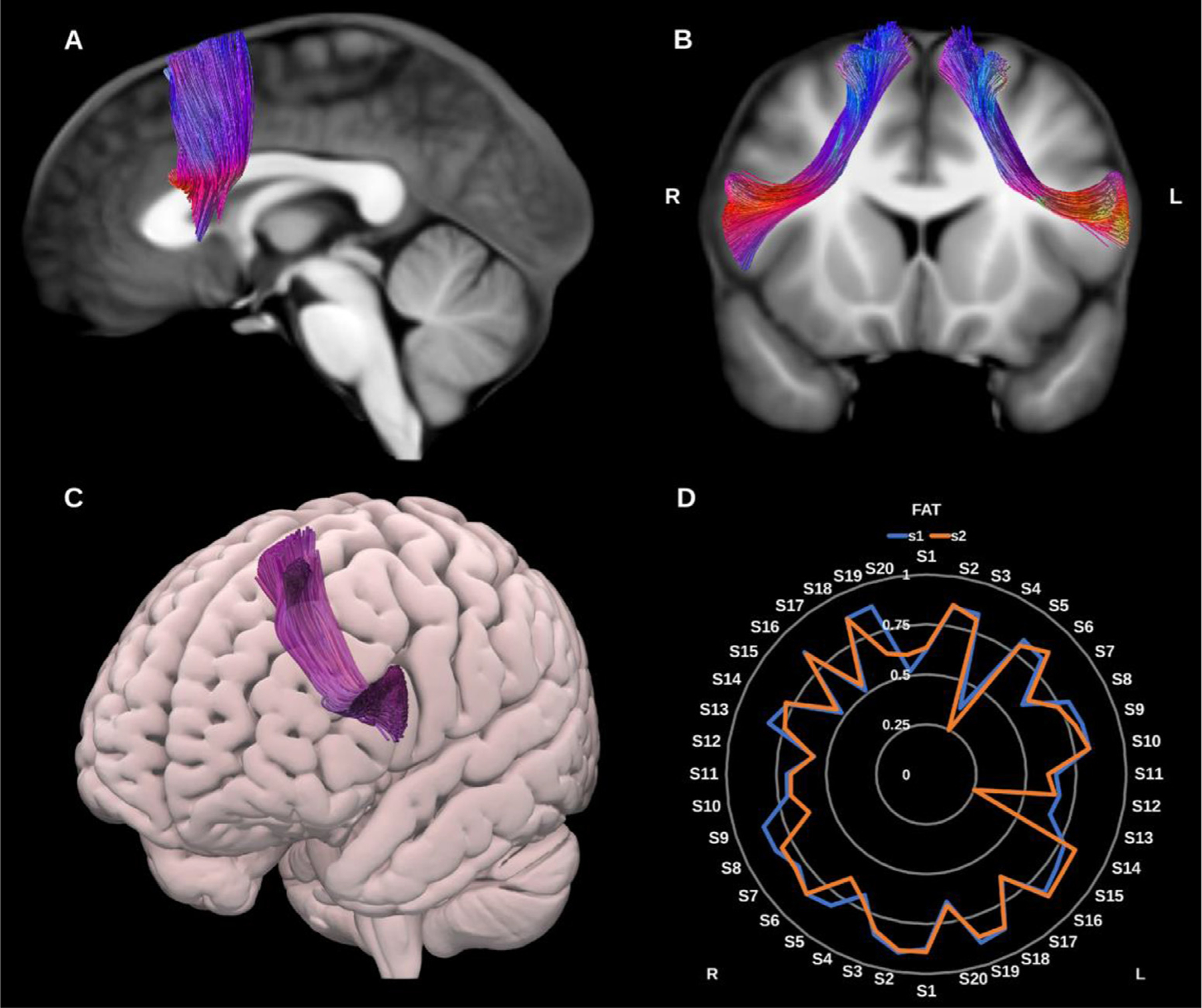
(A) and (B) Frontal aslant tracts (FAT) overlaid in directional color coding on sagittal and coronal slices of the T1-weighted images. (C) 3D oblique anterior projection of the MNI pial surface with left FAT in purple. (D) Radar plot of the wDSC scores (vertical range) of both FATs reconstructed using first session (blue) and second session (orange). L = left, R = right, S = subject. MNI = Montreal Neurological Institute, wDSC = weighted dice similarity coefficient.

The FAT is a recently described association bundle connecting the inferior and superior frontal lobe, and is involved in speech initiation, verbal fluency and executive function/inhibitory control. More specifically it connects the pars opercularis and pars triangularis in the inferior frontal gyrus (IFG) with the pre-supplementary motor area (pre-SMA), supplementary motor area (SMA), and the anterior cingulate cortex (Dick et al., 2019; La Corte et al., 2021; Pascual-Diaz et al., 2020), see Fig. 5.

##### 3.1.1.5. Inferior fronto-occipital fasciculus (IFOF).

**Fig. 6.**
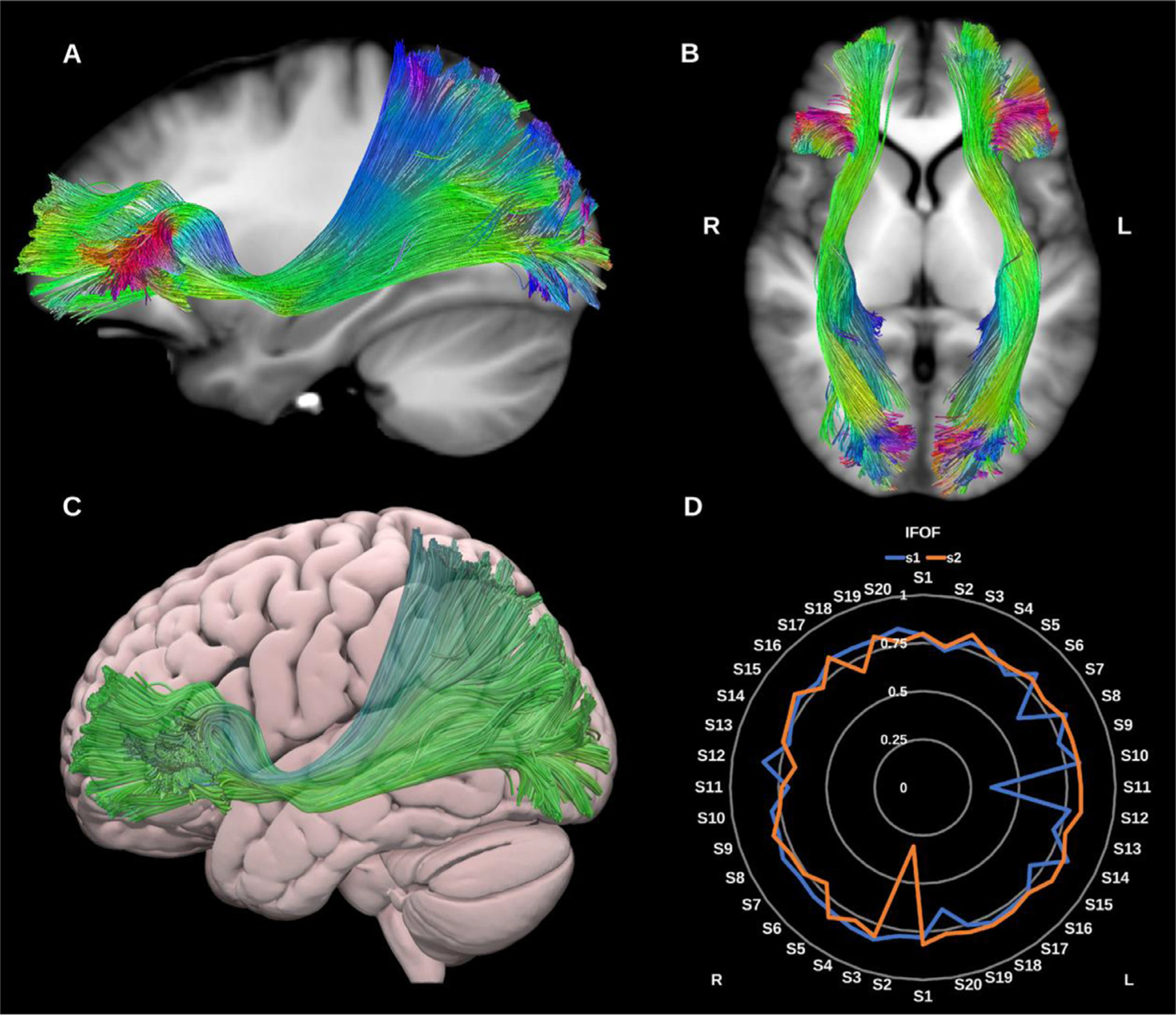
(A) and (B) Inferior fronto-occipital fasciculi (IFOF) overlaid in directional color coding on sagittal and axial slices of T1-weighted images. (C) 3D lateral projection of the semitransparent MNI pial surface with left IFOF in green. (D) Radar plot of the wDSC scores (vertical range) of both IFOFs reconstructed using first session (blue) and second session (orange). L = left, R = right, S = subject, MNI = Montreal Neurological Institute, wDSC = weighted dice similarity coefficient.

The IFOF is a large, long-range association fiber bundle connecting the occipital and temporal lobes to the frontal lobes, specifically the lingual, posterior fusiform, cuneus and polar occipital cortex, with the inferior frontal gyrus, medial fronto-orbital region and frontal pole, see Fig. 6.

Notably, it narrows at the level of the extreme capsule. Though theoretically distinct from other temporal lobe association pathways, the IFOF runs in close proximity to the middle longitudinal fasciculus (MdLF), inferior longitudinal fasciculus (ILF) and uncinate fasciculus (UF), which may pose issues for some tracking algorithms (Forkel et al., 2014; Wu et al., 2016a). Considerable debate prevails regarding its exact functions however it is believed to serve the ventral visual and language streams along with the inferior longitudinal fasciculus (ILF) and uncinate fasciculus (UF) (Caverzasi et al., 2016). The right IFOF may be related to facial recognition functions and semantic visual stream processes (Herbet et al., 2018), while the left IFOF is related to semantic language functions (Almairac et al., 2015).

##### 3.1.1.6. Inferior longitudinal fasciculus (ILF).

**Fig. 7.**
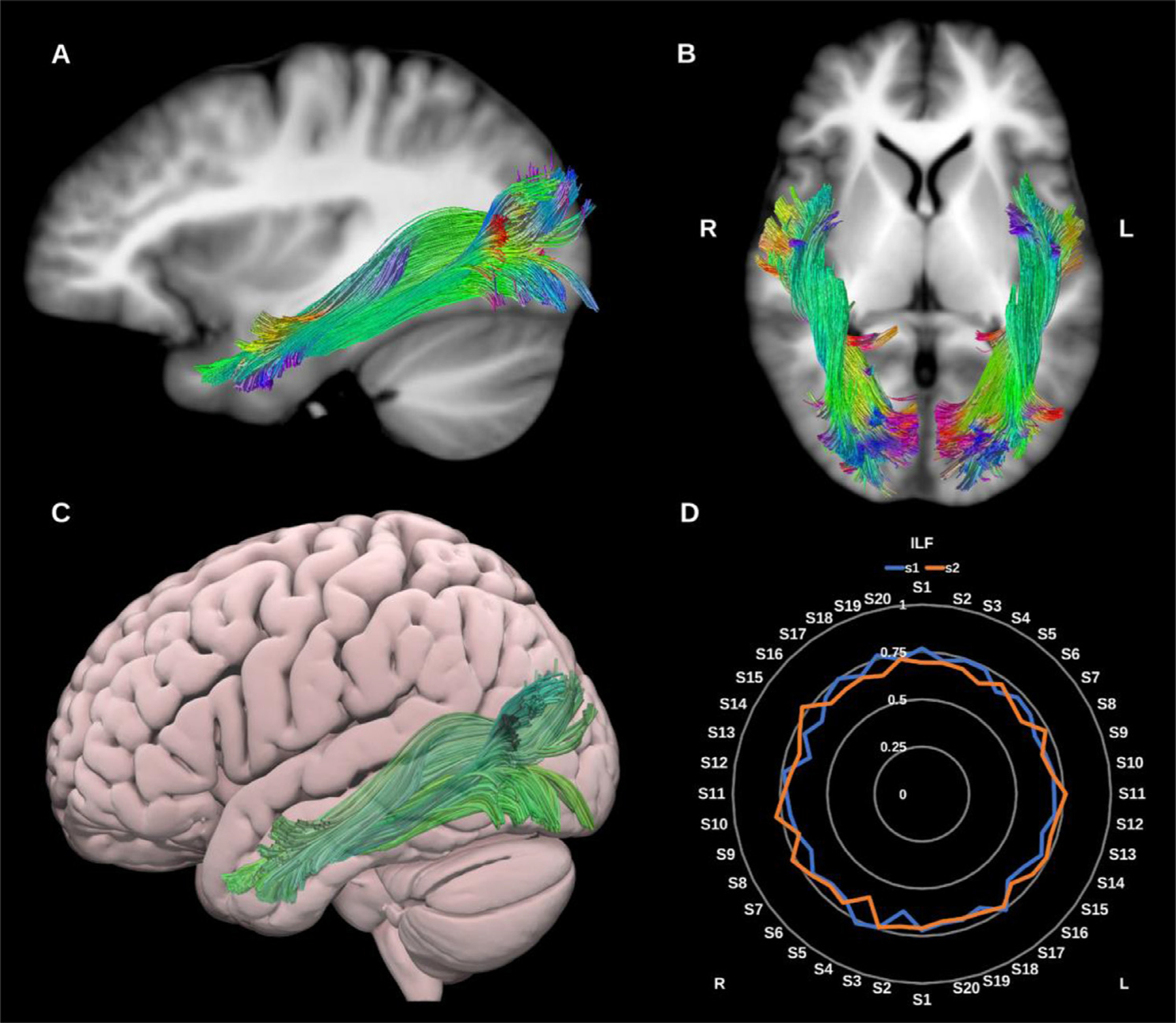
(A) and (B) Inferior longitudinal fasciculi (ILF) overlaid in directional color coding on sagittal and axial slices of the T1-weighted images. (C) 3D lateral projection of the semitransparent MNI pial surface with the left ILF in green. (D) Radar plot of the wDSC scores (vertical range) of both ILFs reconstructed using first session (blue) and second session (orange). L = left, R = right, S = subject, MNI = Montreal Neurological Institute, wDSC = weighted dice similarity coefficient.

The ILF is a large association tract connecting the occipital and temporal lobes and may play an important functional role in visual memory and emotional processing. It lies in direct contact with or close proximity to several bundles, including the UF, IFOF, AF, optic radiations (ORs) and tapetal fibers of the corpus callosum (CC). Whilst commonly depicted as a single bundle, the ILF may have up to four morphological subdivisions which reflect its occipital termination points, and include lingual, cuneate, fusiform and dorso-lateral occipital subcomponents. However, given the lack of consensus about the existence and functional significance of these subdivisions, here we construct the ILF as a single bundle (Herbet et al., 2018; Latini et al., 2017; Panesar et al., 2018), see Fig. 7.

##### 3.1.1.7. Middle longitudinal fasciculus (MdLF).

**Fig. 8.**
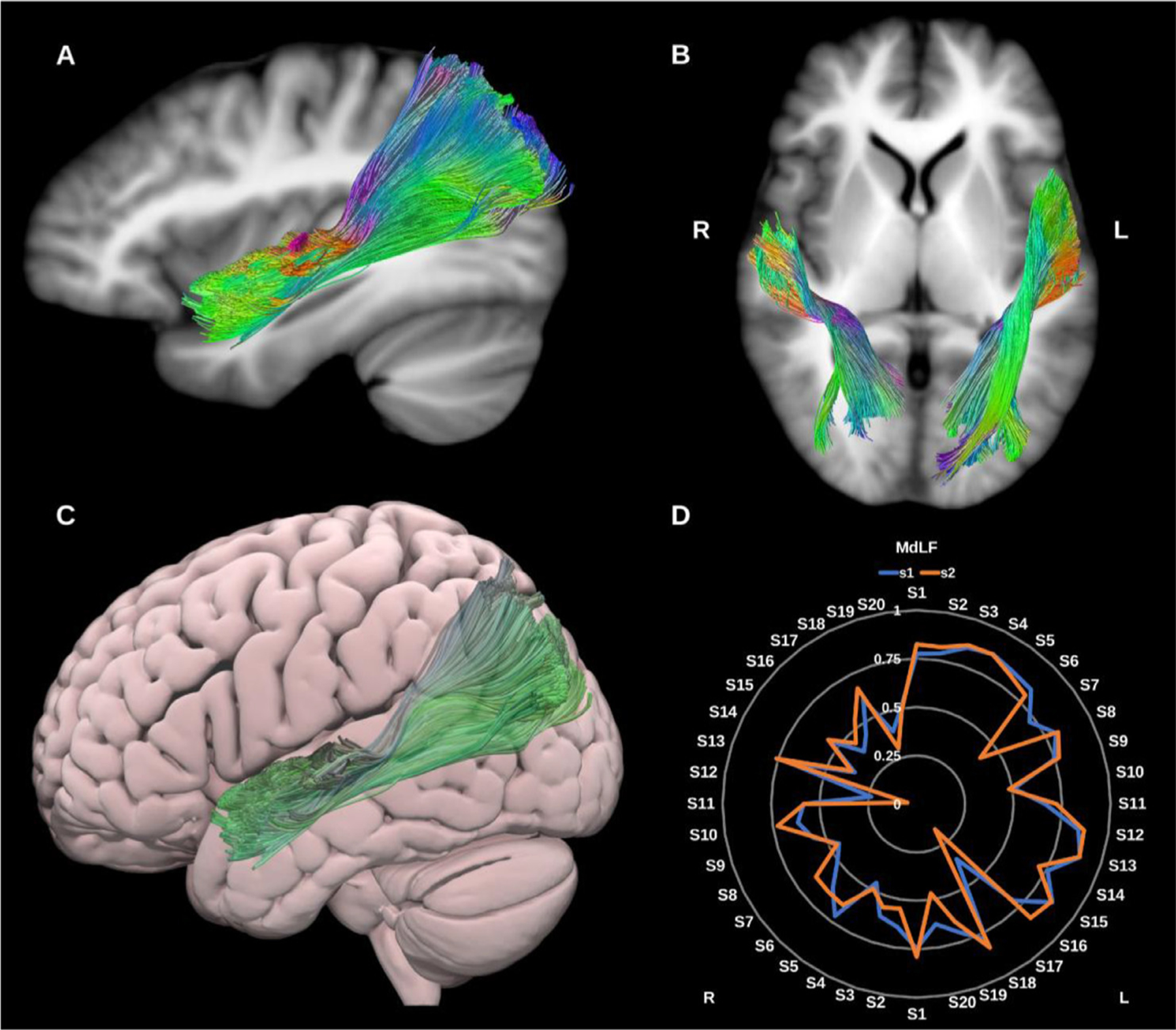
(A), and (B) Middle longitudinal fasciculi (MdLF) overlaid in directional color coding on sagittal and axial slices of the T1-weighted images. (C) 3D lateral projection of the semitransparent MNI pial surface with the left MdLF in green. (D) Radar plot of the wDSC scores (vertical range) of both MdLFs reconstructed using first session (blue) and second session (orange). L = left, R = right, S = subject, MNI = Montreal Neurological Institute, wDSC = weighted dice similarity coefficient.

The MdLF is a large association bundle which is hypothesized to play a role in language, visual and auditory processing (De Witt Hamer et al., 2010; Dick and Tremblay, 2012; Kalyvas et al., 2020). Broadly speaking it is thought to connect the temporal pole, superior temporal gyrus, angular gyrus, superior parietal lobule and precuneus forming a distinct association bundle that runs medial to the AF and lateral to the IFOF (Makris et al., 2017, 2009; Maldonado et al., 2013; Menjot de Champfleur et al., 2013; Seltzer and Pandya, 1984; Wang et al., 2013), see Fig. 8.

##### 3.1.1.8. Superior longitudinal fasciculus (SLF – I, II, and III).

**Fig. 9.**
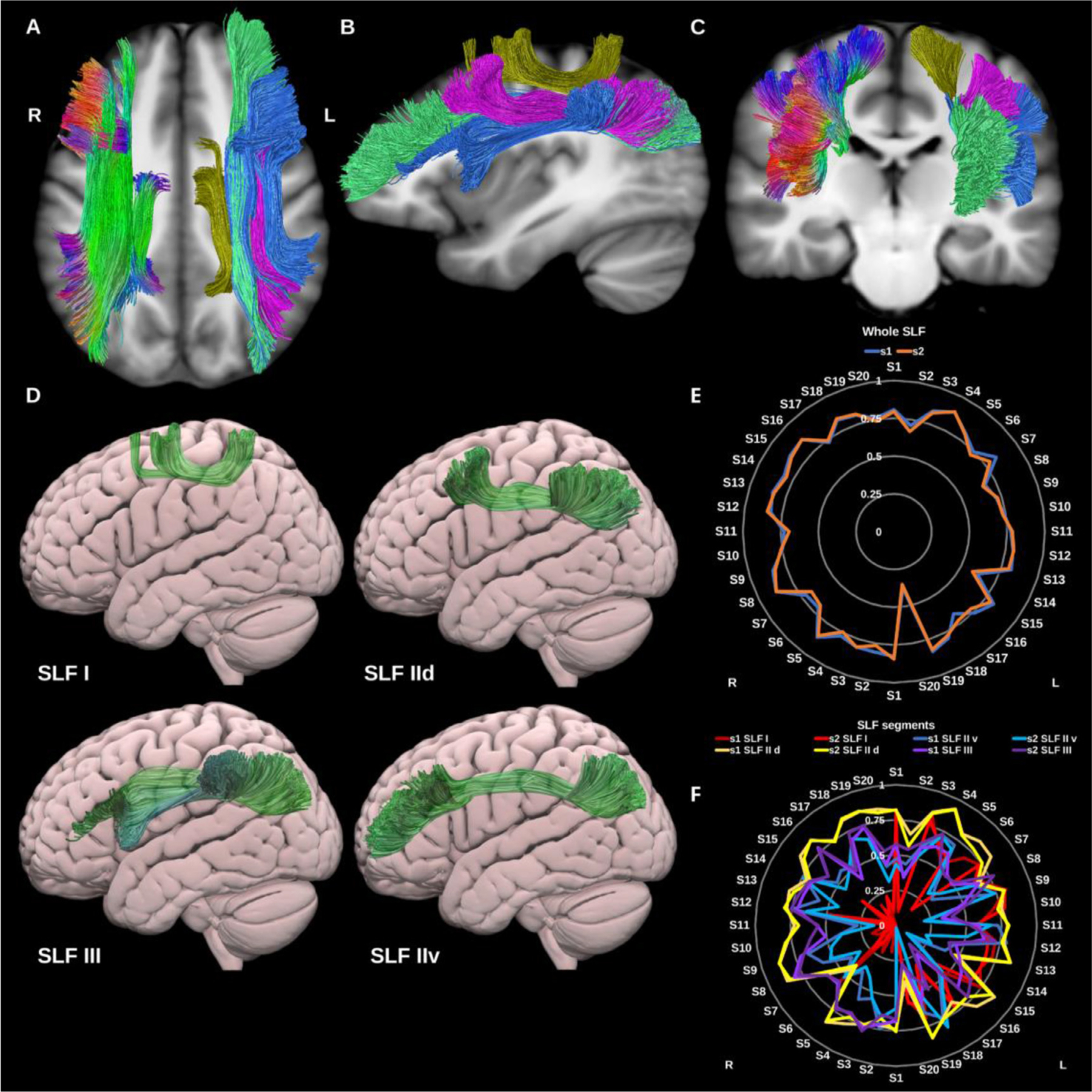
(A), (B), and (C) Superior longitudinal fasciculi (SLF) overlaid in directional color coding on the right side and in gold (SLF-I), pink (SLF-IId), green (SLF-IIv) and blue (SLF-III) on the left side on sagittal and axial slices of T1-weighted images. (D) 3D lateral projections of the semitransparent MNI pial surface with the left SLF I, IId, IIv, and III in green. (E) and (F) Radar plots of the wDSC scores (vertical range) of both whole SLFs (E) reconstructed using first session (blue) and second session (orange), and SLF components (F) for each side. L = left, R = Right, S = subject, MNI= Montreal Neurological Institute, wDSC= weighted dice similarity coefficient.

The SLF is a parieto-occipital association fiber system, located in the dorso-lateral aspect of the cerebrum and generally believed to comprise three or four components (de Schotten et al., 2011; Wang et al., 2016). Broadly speaking it connects the frontal with the occipital, parietal and temporal lobes, see Fig. 9. The SLF is related to language (Madhavan et al., 2014) visuo-spatial (Hong et al., 2019), and meta-cognitive (Zheng et al., 2020) functions. When tracked as a whole the SLF is highly reproducible, but may underestimate the frontal extent of the ventral division of SLF II.

###### 3.1.1.8.1. SLF‐I.

The SLF-I connects the superior parietal lobule and precuneus with posterior superior frontal cortical areas. It is a rather short association bundle that is located above the level of the cingulate cortex (Wang et al., 2016). This was generated for all datasets on both sides but was the least reproducible of the SLF segments, most likely due to its small volume and low streamlines count.

###### 3.1.1.8.2. SLF‐II.

SLF-II is located more infero-lateral to SLF-I and originates in the anterior intraparietal sulcus and the angular gyrus, terminating in the posterior regions of the superior and middle frontal gyri. SLF-II can be subdivided into two distinct subcomponents (Barbeau et al., 2020), the first is the dorsal component (SLF-IId), which connects the supramarginal and inferior parietal cortices to the dorsal middle frontal gyrus. The second is the ventral component (SLF-IIv), which is longer, more ventral and connects the supramarginal and inferior parietal cortices to the rostral middle frontal gyrus.

###### 3.1.1.8.3. SLF‐III.

SLF-III connects the intraparietal sulcus and inferior parietal lobule to the inferior frontal gyrus (Barbeau et al., 2020). It is immediately superior and medial to the arcuate fasciculus partially overlapping with the horizontal fibers of the Arcuate fasciculus.

##### 3.1.1.9. Uncinate fasciculus (UF).

**Fig. 10.**
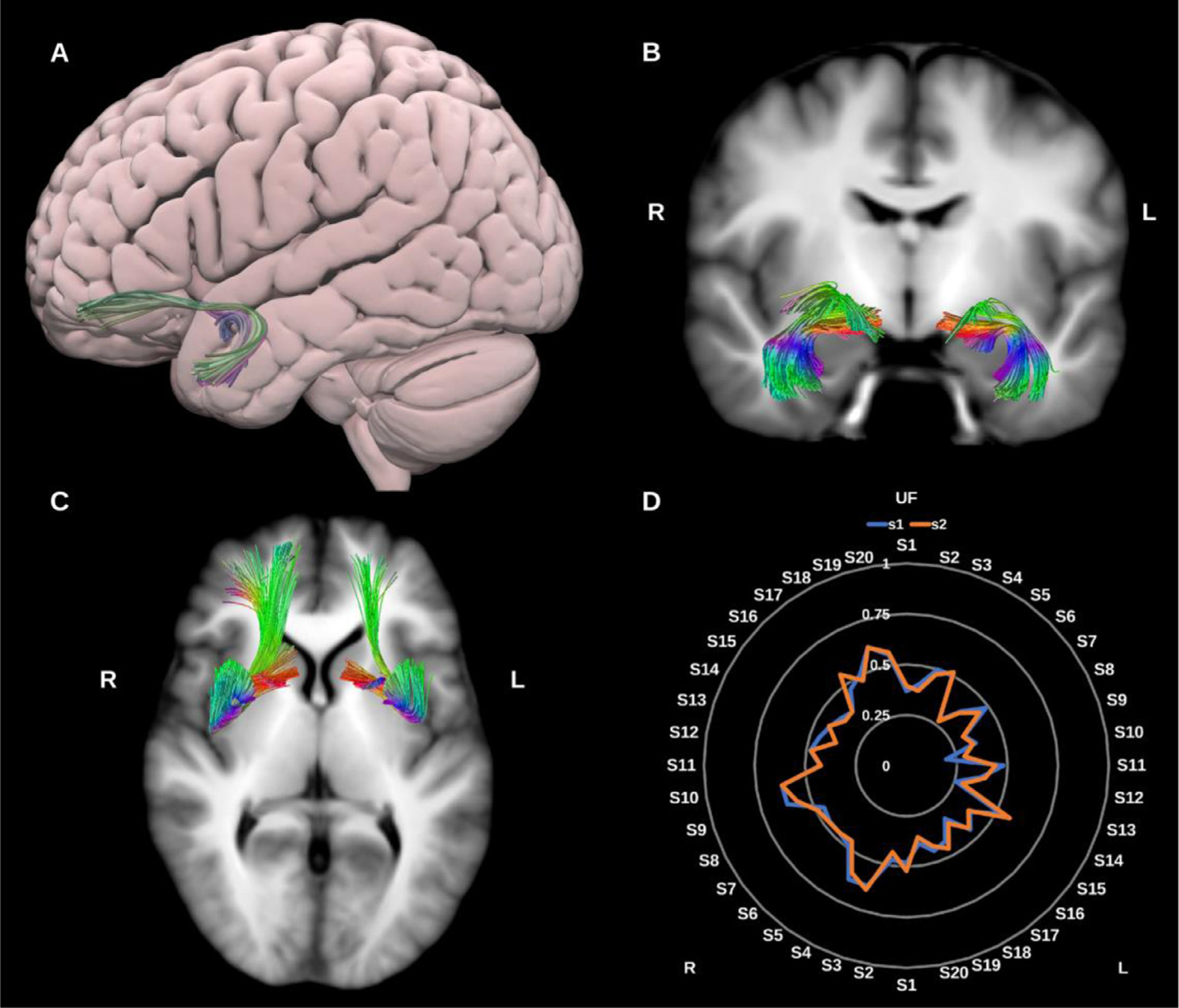
(A) 3D lateral projection of the semitransparent MNI pial surface with the left uncinate fasciculus (UF) shown in directional color coding. (B) and (C) Both UFs overlaid in directional color coding on coronal and axial slices of the T1-weighted images. (D) Radar plot of the wDSC scores (vertical range) of both UFs reconstructed using first session (blue) and second session (orange). L = left, R = right, S = subject, MNI = Montreal Neurological Institute, wDSC = weighted dice similarity coefficient.

The UF is typically characterized as a hook shaped bidirectional association fiber bundle linking the ventral, medial and orbital frontal lobes and rostral temporal lobes. Whilst DTI typically reconstructs a short hook-shaped fasciculus (Kurki et al., 2013), higher order dMRI reconstructions have revealed a more extensive fiber system which expands into a fan shaped trajectory in the frontal lobe. Such higher order models (e.g., CSD) in combination with microdissection suggest that the UF may be further subdivided into 5 subcomponents (Hau et al., 2017). However, for the present reconstruction, we opted to reconstruct the UF as one bundle for practicality, see Fig. 10.

##### 3.1.1.10. Vertical occipital fasciculus (VOF).

**Fig. 11.**
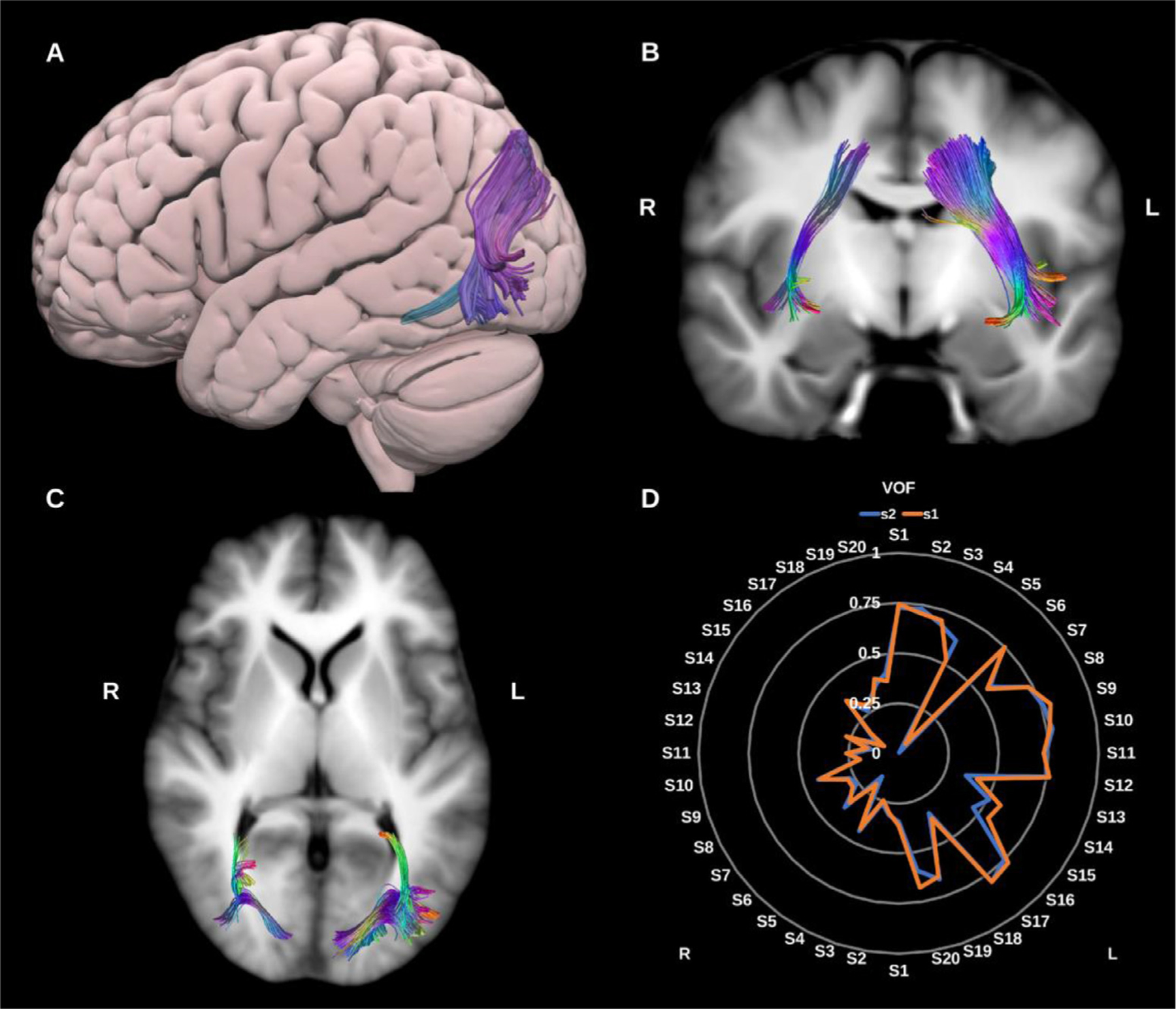
(A) 3D lateral projection of the semitransparent MNI pial surface with the left vertical occipital fasciculus (VOF) in directional color coding, (B) and (C) Both VOFs overlaid in directional color coding on coronal and axial slices of the T1-weighted images. (D) Radar plot of the wDSC scores (vertical range) of both VOFs reconstructed using first session (blue) and second session (orange). L = left, R = right, S = subject, MNI = Montreal Neurological Institute, wDSC = weighted dice similarity coefficient.

The VOF is described as a short slanted vertical association bundle in the lateral aspect of the occipital lobe. It connects the superior part of the occipital lobe and adjacent cortex of the occipito-parietal sulcus to the inferior aspect of the occipital lobe and adjacent occipito-temporal cortical areas (Jitsuishi et al., 2020; Schurr et al., 2019; Yeatman et al., 2014), see Fig. 11.

#### 3.1.2. Commissural fiber bundles

##### 3.1.2.1. Anterior commissure (AC).

**Fig. 12.**
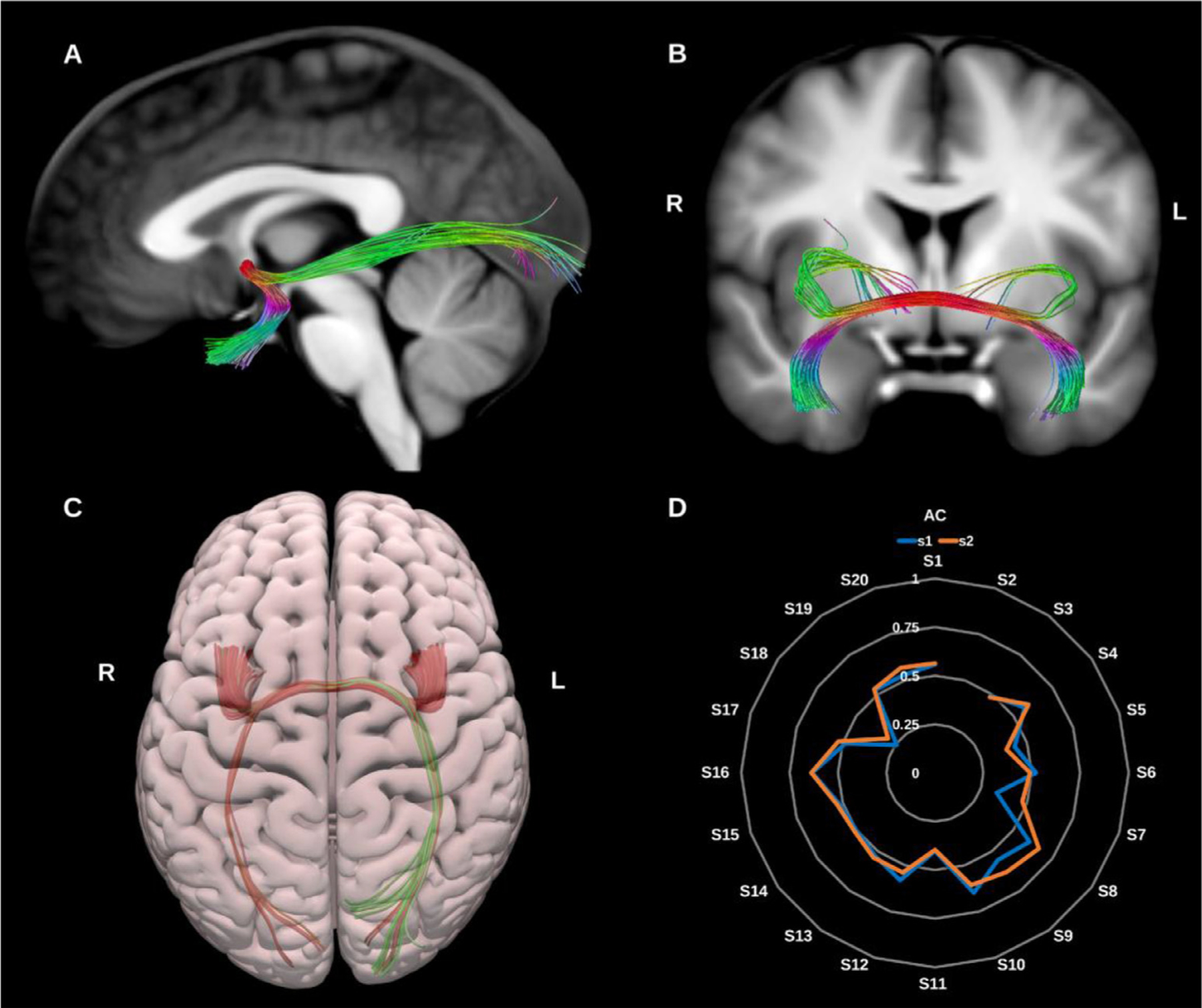
(A) and (B) Anterior commissure (AC) overlaid in color coding on sagittal and coronal slices of the T1-weighted images. (C) 3D superior projection of the semitransparent MNI pial surface with the AC shown in directional color coding. (D) Radar plot of the wDSC scores (vertical range) of the AC reconstructed using first session (blue) and second session (orange). L = left, R = right, S = subject, MNI = Montreal Neurological Institute, wDSC = weighted dice similarity coefficient.

The anterior commissure crosses the midline anterior to the pre-commissural columns of the fornix, above the basal forebrain and below the medial and ventral portion of the anterior limb of the internal capsule. It has two main parts, the first, anterior division which includes the olfactory decussation and the second, largest division which connects the temporal lobes, occipital lobes, and pre-dominantly the bilateral amygdalae (van Meer et al., 2016). During its course the AC intersects with the UF, ILF, sagittal stratum and optic radiations, which complicates tracking the true extent of the structure (Çavdar et al., 2021; Choi et al., 2011; Kikinis et al., 2015; Peltier et al., 2011; van Meer et al., 2016; Wilde et al., 2006), see Fig. 12.

##### 3.1.2.2. Corpus callosum (CC).

**Fig. 13.**
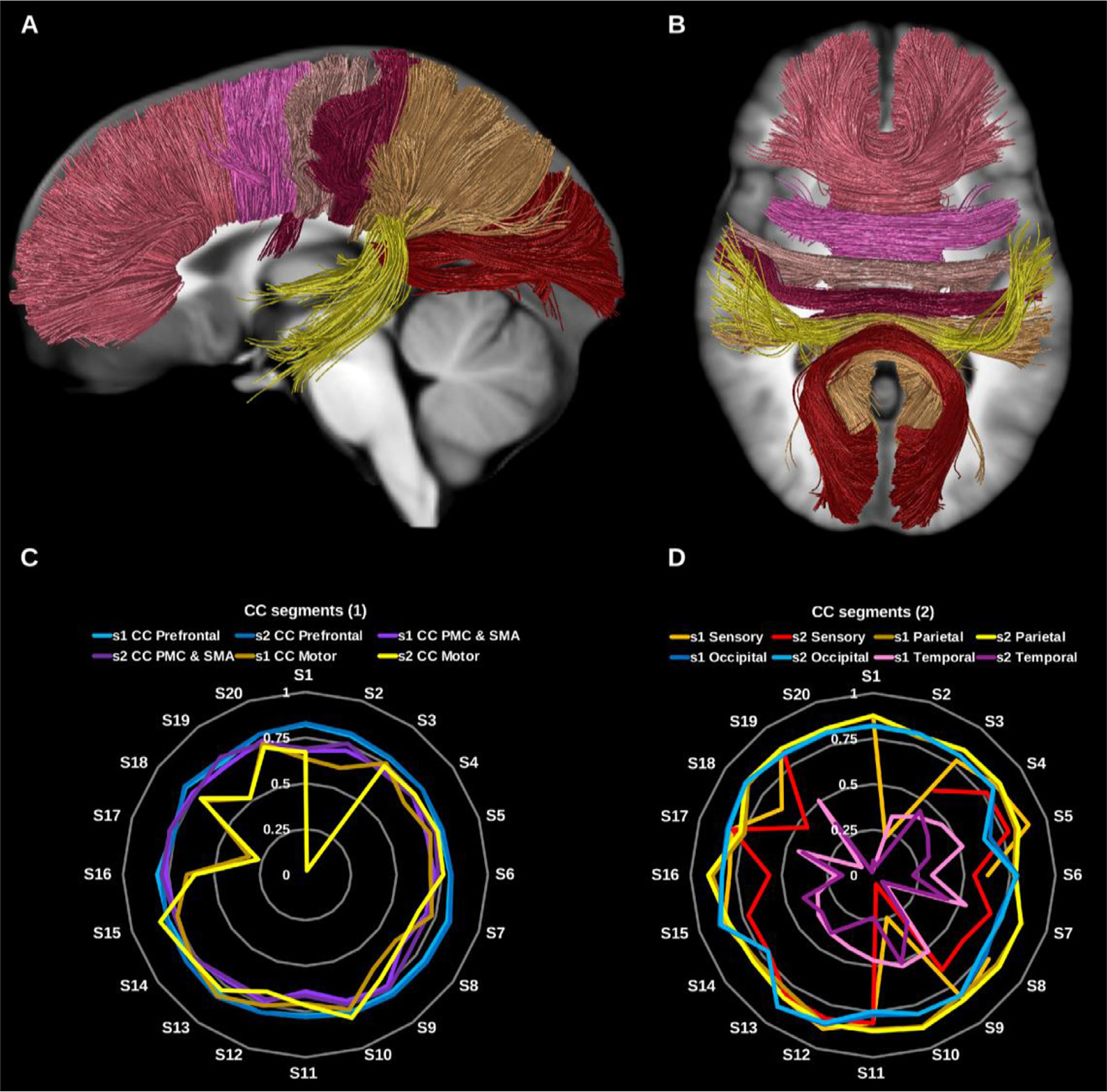
(A) and (B) Corpus callosum (CC) segments shown in solid colors per segment on sagittal and axial slices of the T1-weighted images. (C) and (D) Radar plots of wDSC (vertical ranges) resulting from comparison to HCP-template bundles (C) prefrontal CC, premotor and supplementary motor CC, and motor CC, (D) sensory CC, parietal CC, occipital CC, and temporal CC. PMC = premotor cortex, SMA = supplementary motor cortex, wDSC = weighted dice similarity coefficient.

**Fig. 14.**
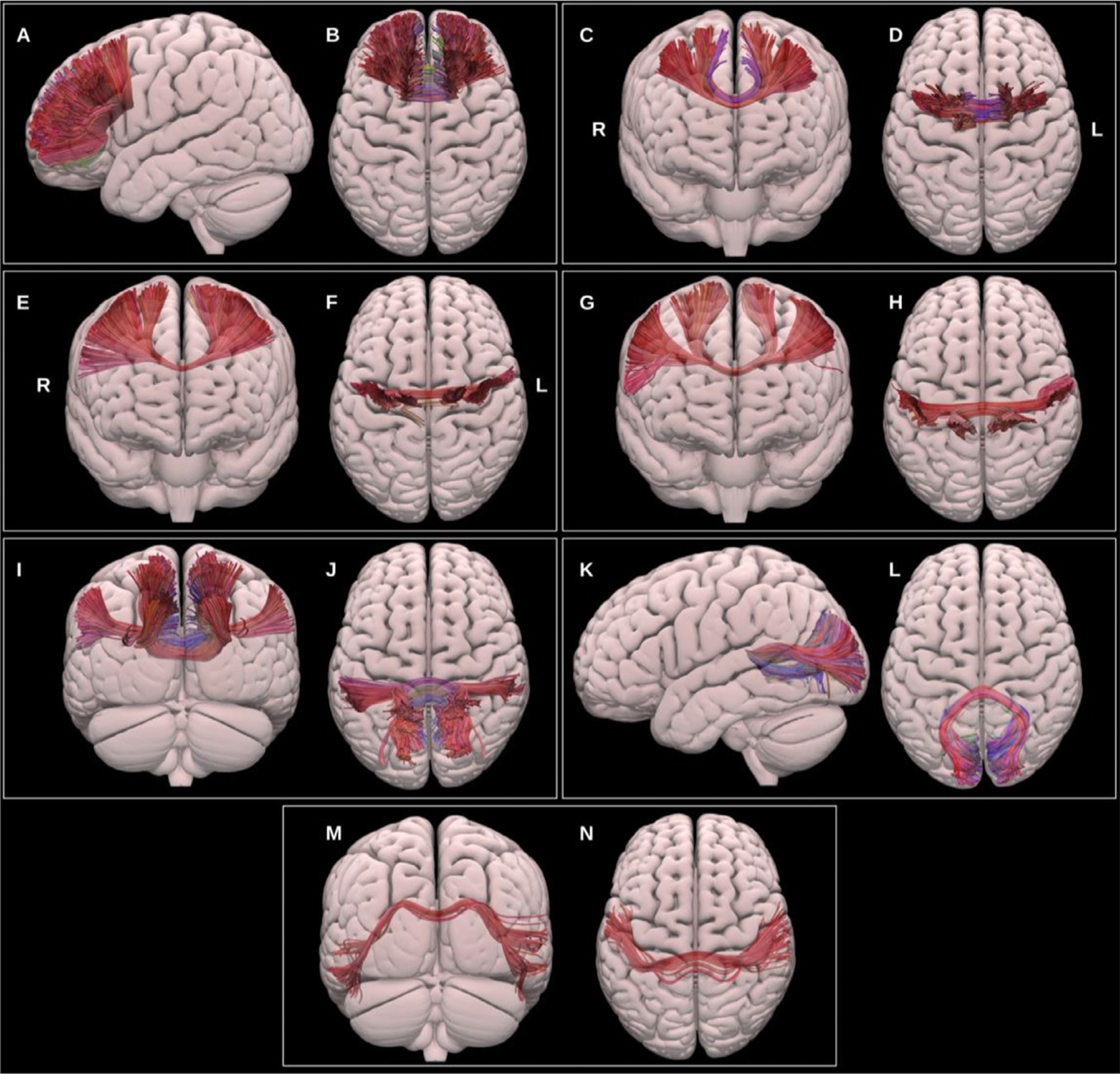
Multiple projections of the semitransparent MNI pial surface, with lateral and superior views showing the prefrontal CC (A & B), anterior and superior views showing the PMC and SMA CC (C & D), anterior and superior views showing the motor CC (E & F), anterior and superior views showing the sensory CC (G & H), posterior and superior views showing the parietal CC (I & J), lateral and superior views showing the occipital CC, and posterior and superior views showing the temporal CC (M & N).

The corpus callosum is the largest fiber bundle in the human brain and connects the right and left cerebral hemispheres, hence its primary function is interhemispheric information transfer and integration. Traditionally the CC is divided into several subcomponents: the rostrum, genu, body, isthmus, splenium and tapetum. Here we reconstructed the CC in 7 segments comprising the prefrontal, premotor, motor, sensory, parietal, occipital and temporal callosal fibers, see Figs. 13 and 14.

It is common in DTI based literature to find three subdivisions: the genu, forming the forceps minor and connecting left and right prefrontal and anterior cingulate cortices; the callosal body, and the splenium, which forms the forceps major and connects left and right posterior parietal, medial occipital and medial temporal cortices. Improvements in fiber-tracking algorithms and integration with functional data have led to alternative parcellation strategies (Catani, 2006; Fabri et al., 2014; Nazem-Zadeh et al., 2012; Ohoshi et al., 2019, 2019; Phillips et al., 2013).

###### 3.1.2.2.1. Prefrontal corpus callosum (CC PreF).

This was reconstructed for all datasets using the bilateral prefrontal cortices and the genu and anterior third of the body of the corpus callosum.

###### 3.1.2.2.2. Premotor and supplementary motor corpus callosum (CC PMC and SMA).

This was reconstructed for all datasets using the caudal middle frontal gyri, the supplementary motor areas bilaterally and the central body of the corpus callosum.

###### 3.1.2.2.3. Motor corpus callosum (CC motor).

This segment connects the primary motor cortices (precentral gyri) of both hemispheres via the posterior third of the body of the corpus callosum. The motor CC was generated for all datasets.

###### 3.1.2.2.4. Sensory corpus callosum (CC sensory).

This was reconstructed using the primary sensory cortices (postcentral gyri) on both sides via the posterior third of the body and splenium of the corpus callosum in the midline. The sensory CC was reconstructed for all except 4 datasets.

###### 3.1.2.2.5. Parietal corpus callosum (CC parietal).

This was reconstructed for all data using the whole parietal lobes bilaterally and the splenium of the corpus callosum.

###### 3.1.2.2.6. Occipital corpus callosum (CC occipital).

This was reconstructed for all data using the occipital lobes on both sides and the splenium of the corpus callosum.

###### 3.1.2.2.7. Temporal corpus callosum (CC temporal).

This was reconstructed using the lateral aspect of the temporal lobes bilaterally and the splenium of the corpus callosum in the midline. The temporal CC reconstruction failed in 1 dataset.

#### 3.1.3. Projection fiber bundles

##### 3.1.3.1. Medial lemniscus (ML).

**Fig. 15.**
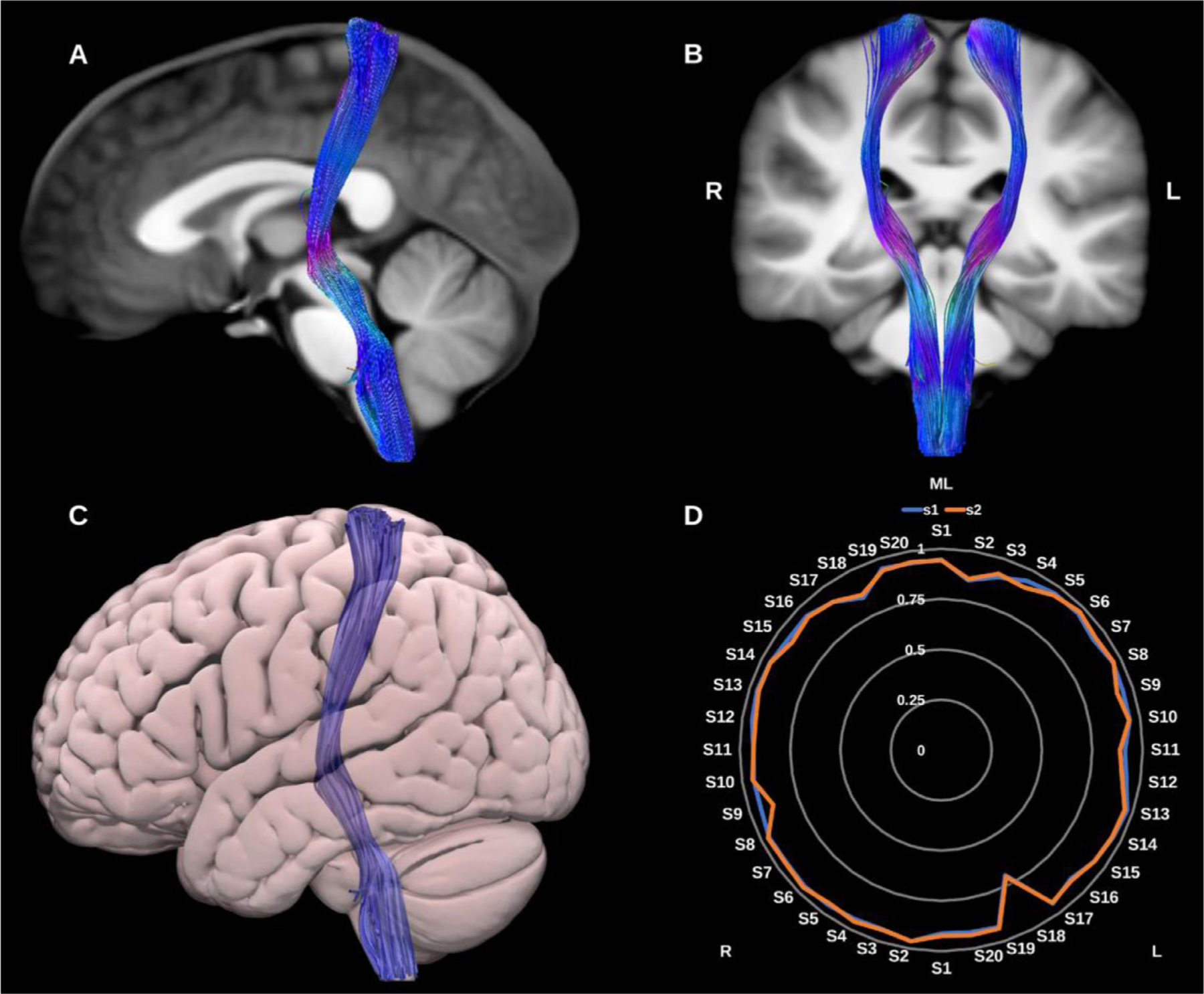
(A) and (B) Medial lemniscus (ML) overlaid in directional color coding on sagittal and coronal slices of the T1-weighted images. (C) 3D lateral projection of the semitransparent MNI pial surface with the ML in blue. (D) Radar plot of the wDSC scores (vertical range) of the ML reconstructed using first session (blue) and second session (orange). L = left, R = right, S = subject, MNI = Montreal Neurological Institute, wDSC = weighted dice similarity coefficient.

The primary dorsal ascending tracts originate in the dorsal columns of the spinal cord, ascend through the medulla oblongata to the thalamic ventral-posterior medial (VPM) and lateral (VPL) nuclei and via the internal capsule to the primary somatosensory cortex in the postcentral gyrus (Jang and Seo, 2015; Peng et al., 2019a), see Fig. 15. The ML was reconstructed using the UKBB-derived posterior brainstem VOIs, the VPM and VPL thalamic nuclei, and primary sensory cortices.

##### 3.1.3.2. Optic radiation (OR).

**Fig. 16.**
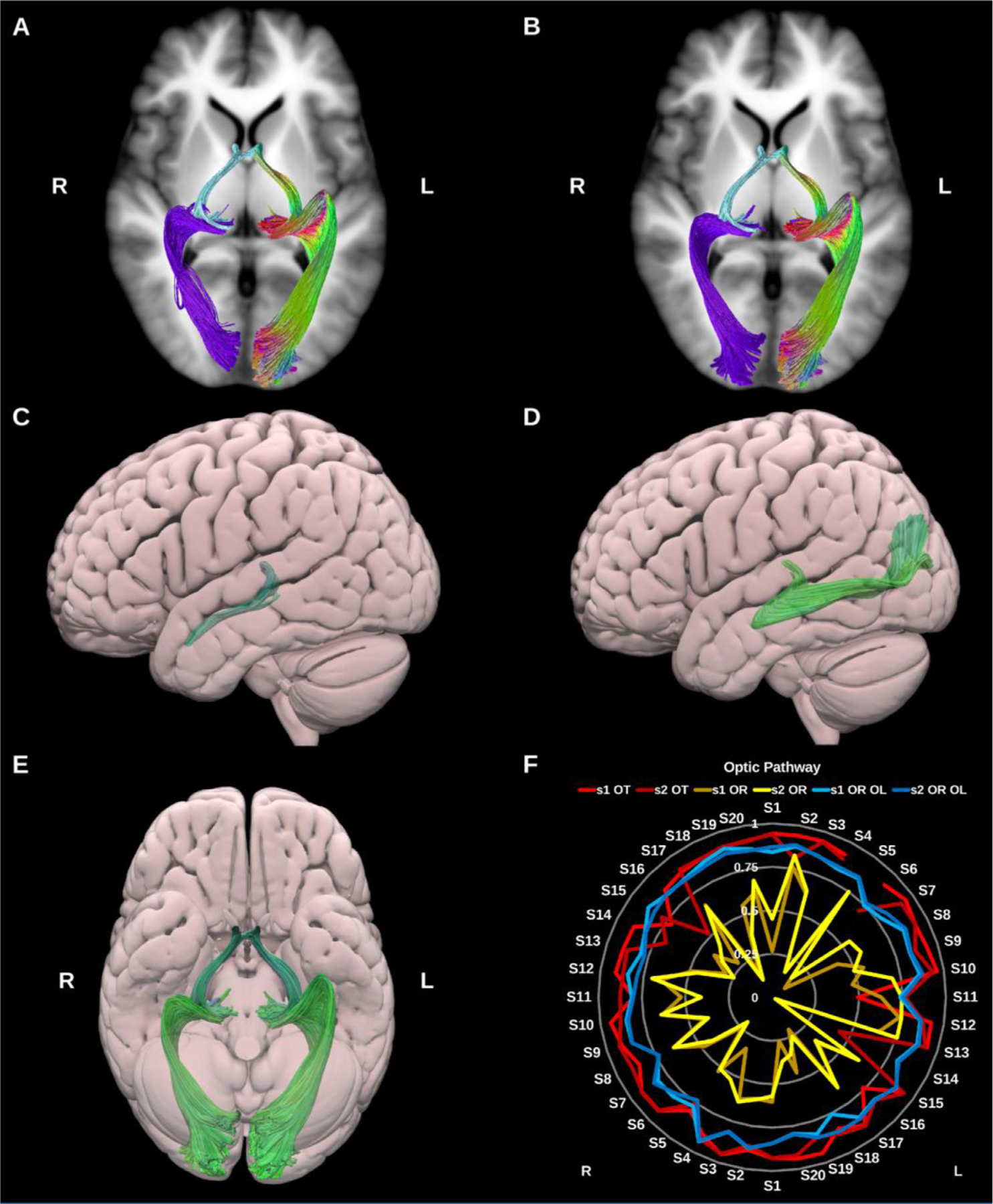
(A) and (B) Optic pathway bundles overlaid on axial slices of the T1-weighted images. The optic tracts (OT) (light blue) and (A) classic optic radiation (OR) (purple), and (B) whole occipital lobe optic radiations (OR OL) (purple) on the right side, and directional color coding on the left side. (C) and (D) 3D lateral projections of the semitransparent MNI pial surface with the OT (C) and optic radiation (D). (E) 3D inferior projection for the entire optic pathway on both sides. (F) Radar plot of the wDSC scores (vertical range) of the optic tracts and both versions of the optic radiations. L = left, R = right, S = subject, MNI = Montreal Neurological Institute, wDSC = weighted dice similarity coefficient.

The ORs connect the lateral geniculate nucleus of the thalamus to the primary visual cortex within the occipital lobe, see Fig. 16. They are critical for visual processing and present important challenges during surgical procedures involving the temporal lobe. Notably, Meyer’s loop presents a particular challenge for tractography (Mehra and Moshirfar, 2021). This portion of the OR, which exhibits significant inter-individual variability, is characterized by a sharp anterior projection in the anterior temporal lobe that then bends posteriorly to join the sagittal stratum. Moreover, within the anterior loop, OR fibers may intersect with fibers of the anterior commissure, ILF and tapetum, increasing the likelihood of spurious streamlines (Chamberland et al., 2018; Goga and Türe, 2015; Lim et al., 2015; Martínez-Heras et al., 2015). We generated two representations of the optic radiations, first using a classical anatomical definition with only the pericalcarine cortex as a cortical inclusion VOI, and second using the entire occipital lobe as a cortical inclusion VOI.

##### 3.1.3.3. Optic tract (OT).

The OT is formed of decussating axons from the contra-lateral optic nerve as well as non-decussating fibers from the ipsilateral optic nerve. It extends from the optic chiasm to the lateral geniculate nucleus of the thalamus (Hofer et al., 2010; Mehra and Moshirfar, 2021; Wu et al., 2012).

##### 3.1.3.4. Pyramidal tract (PyT).

**Fig. 17.**
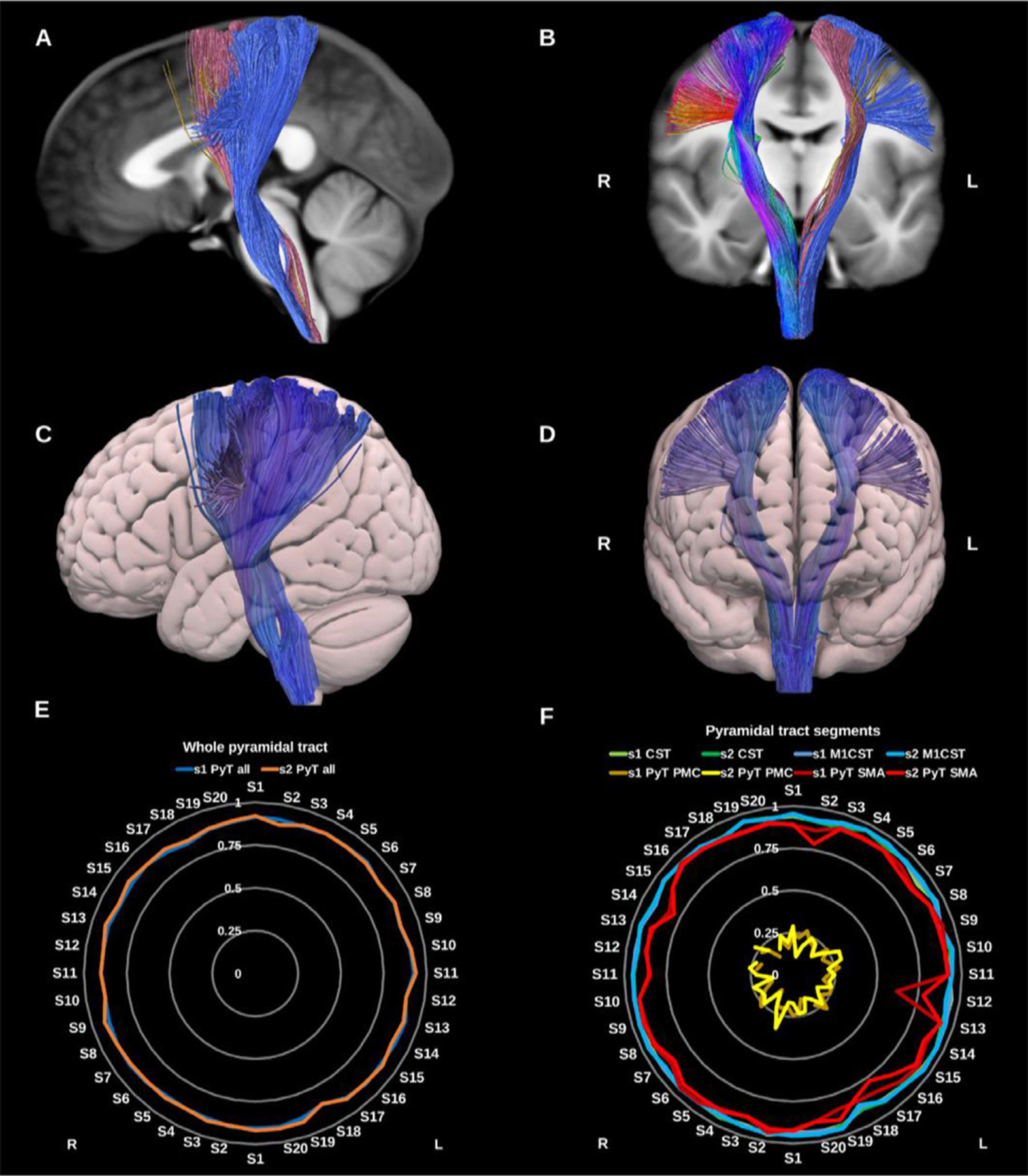
(A) and (B) whole pyramidal tract (PyT_all) overlaid in directional color coding on the right side and its different components on the left side in solid colors, premotor pyramidal tract (PyT_PMC) in yellow, supplementary motor area pyramidal tract (PyT_SMA) in pink and the corticospinal tract (CST) in blue on sagittal and coronal slices of the T1-weighted images. (C) and (D) 3D lateral and anterior projections of the semitransparent MNI pial surface with the whole PyT on both sides shown in blue. Radar plots of wDSC (vertical ranges) resulting from comparison to HCP-template bundles are shown in (E) for the whole PyT, and in (F) for the different pyramidal tract segments. L = left, R = right, S = subject, MNI = Montreal Neurological Institute, wDSC = weighted dice similarity coefficient, M1 CST = motor only CST.

The pyramidal tract fibers carry motor impulses from the cerebral cortex to the spinal cord through the brainstem. Here we reconstruct the Pyramidal tract (PyT) as a whole including the primary motor and sensory cortices along with the premotor, supplementary motor and parietal proprioceptive cortices superiorly and the whole brain stem inferiorly. We also provide dissections of this bundle based on specific sub-systems of the sensory-motor network (Chenot et al., 2019; Jeong et al., 2013; Peng et al., 2019b; Zhang et al., 2010), see Fig. 17.

###### 3.1.3.4.1. Primary sensory-motor pyramidal tract (Corticospinal tract) (CST).

The CST descends predominantly from the primary motor areas of the precentral gyrus and somatosensory areas of the postcentral gyrus through the corona radiata, posterior half of the posterior limb of the internal capsule, and cerebral peduncles to the rostral brainstem where it forms the medullary pyramids before crossing the pyramidal decussation on its further descent through the spinal cord. We provide two reconstructions of this bundle, one using the primary motor and sensory cortices (CST) and another using only the primary motor cortices, excluding the postcentral gyri (M1_CST).

###### 3.1.3.4.2. Premotor pyramidal tract (PyT PMC).

This represents the pyramidal tract fibers originating in the dorsal premotor cortex (caudal middle frontal gyrus) and descending to the brainstem via the anterior half of the posterior limb of the internal capsule. This bundle had the lowest number of streamlines and was the least reproducible of the pyramidal tract components.

###### 3.1.3.4.3. Supplementary motor pyramidal tract (PyT SMA).

This represents the pyramidal tract fibers originating in the supplementary motor area and descending to the brainstem via the centrum semiovale and the anterior half of the posterior limb of the internal capsule.

##### 3.1.3.5. Thalamic radiations (TRs).

**Fig. 18.**
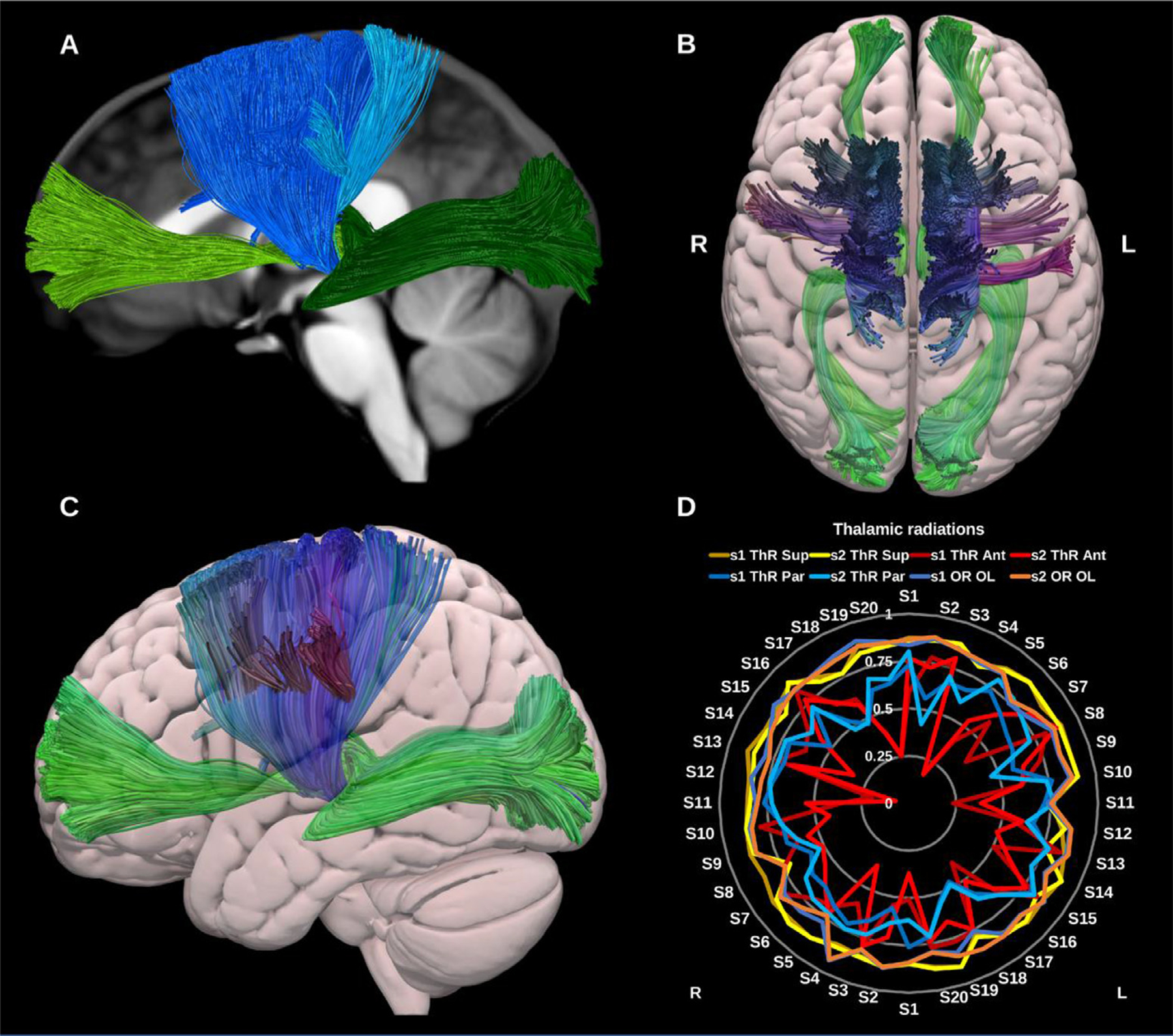
(A) Thalamic radiations overlaid in solid colors on a midline sagittal slice of the T1-weighted image. The anterior thalamic radiation (ATR) is shown in light green, the superior thalamic radiation (STR) in blue, the parietal thalamic radiation (PaTR) in turquoise, and the posterior thalamic radiation in dark green (OR OL). (B) and (C) 3D superior and lateral projections of the semitransparent MNI pial surface with the ATR and OR OL in green, and the STR and PaTR in blue. (D) Radar plot of the wDSC scores (vertical range) of the different thalamic radiations. L = left, R = right, S = subject, MNI = Montreal Neurological Institute, wDSC = weighted dice similarity coefficient, OR OL = occipital thalamic radiation (same as Optic radiation to whole occipital lobe).

The thalamic radiations are a group of 4–5 thalamo-cortical projection bundles comprising both afferent and efferent fibers. Collectively, the fibers of the thalamic radiations can be described as fanning out towards the cortex and banding up as they approach the thalami. As they run through the internal capsule and corona radiata they intersect with several other projection bundles, which in addition to the complex nature of thalamic microanatomy, can complicate reconstruction (Behrens et al., 2003; Kakou et al., 2017; Lyness et al., 2014; Tsao et al., 2015; Younes et al., 2019), see Fig. 18.

The posterior thalamic radiation is represented by the extended version of the optic radiation (OR OL) including the entire occipital cortex. All thalamic radiations were reconstructed using the whole thalamus as an inclusion VOI and excluding non-contributing thalamic nuclei, e.g., the VPM and VPL nuclei were excluded for the anterior thalamic radiation.

###### 3.1.3.5.1. Anterior thalamic radiation (ATR).

The ATR connects the dorso-medial (DM), dorso-lateral (DL) and anterior thalamic (ATN) nuclei to the prefrontal cortex and is thought to be involved in executive functions and complex planning (Niida et al., 2018). The ATR was reconstructed using the thalamus excluding all nuclei but the DM, DL, and ATN, and the ipsilateral medial and rostral prefrontal cortices.

###### 3.1.3.5.2. Superior thalamic radiation (STR).

The STR connects the ventral thalamic nuclei to the motor and sensory cortices via the superior thalamic peduncle, posterior limb of internal capsule, and the corona radiata, conducting cerebellar and basal ganglia input to the motor cortex (Bosch-Bouju et al., 2013). The STR was reconstructed using the thalamus, excluding the dorso-medial, dorso-lateral and pulvinar nuclei, and the ipsilateral primary motor, supplementary motor and dorsal premotor cortices (Younes et al., 2019).

###### 3.1.3.5.3. Parietal thalamic radiation (PaTR).

This was reconstructed using the entire parietal lobe as a cortical include and the thalamus excluding all except the VPM, and VPL nuclei. This resulted in a bundle of streamlines connecting the thalamic VPM and VPL predominantly to the primary sensory cortex.

###### 3.1.3.5.4. Posterior thalamic radiation (PoTR/OR OL).

This bundle was considered to be the same as the extended optic radiation using the entire occipital lobe, and was not reconstructed separately but will be added in future versions.

#### 3.1.4. Cerebellar bundles

##### 3.1.4.1. Dentato‐rubro‐thalamic tract (DRTT).

**Fig. 19.**
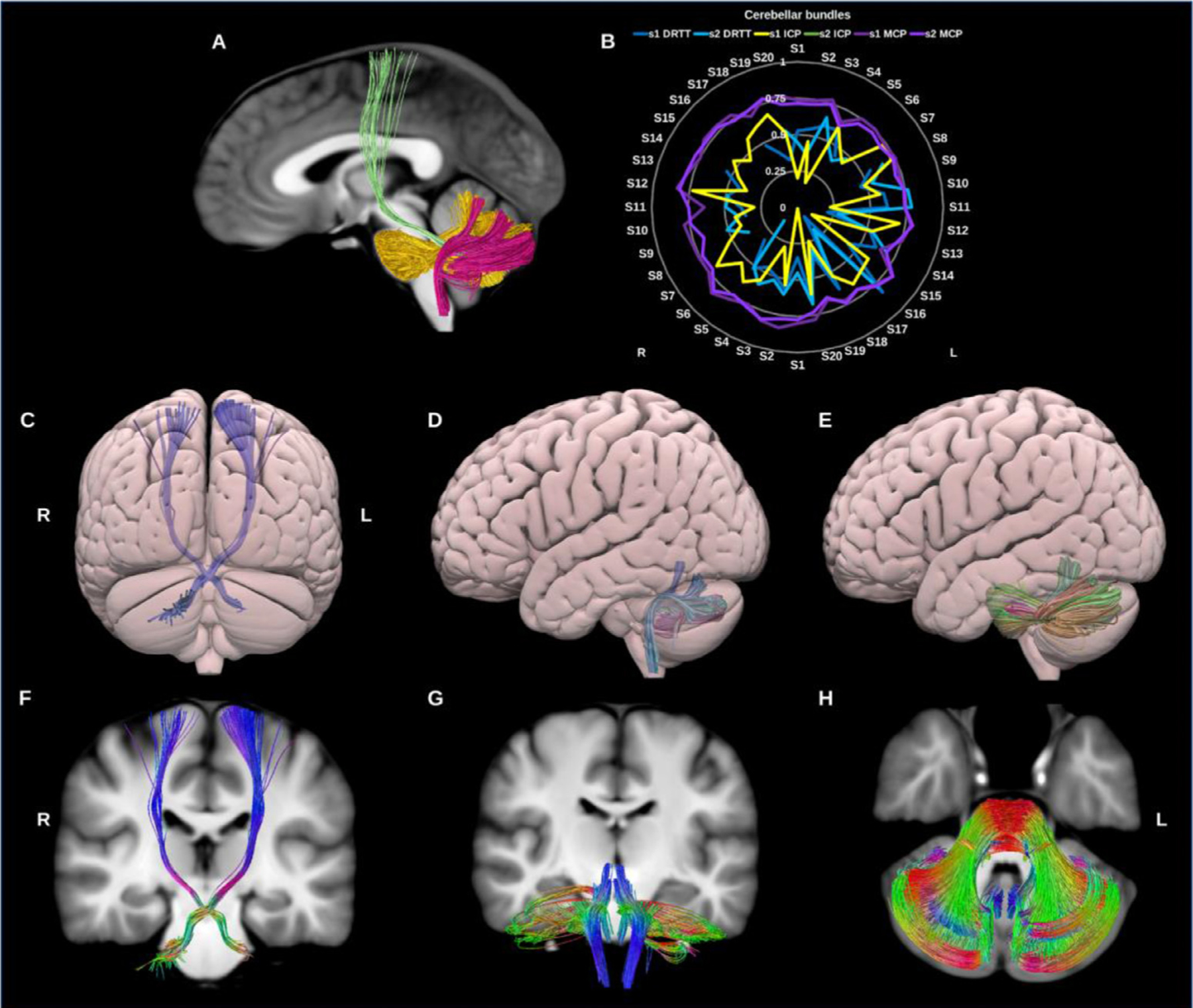
(A) Cerebellar bundles overlaid on sagittal slice of the T1-weighted images. The dentato-rubro-thalamic tract (DRTT) in green, inferior cerebellar peduncle (ICP) in fuschia, and in gold the middle cerebellar peduncle (MCP). (B) Radar plots of wDSC (vertical range) for these three bundles per session. (C), (D) & (E) show posterior and lateral surface views of the DRTT, ICP and MCP. (F), (G) & (H) show T1 coronal and axial slices of the DRTT, ICP and MCP. L = left, R = right, S = subject, wDSC = weighted dice similarity coefficient, MNI = Montreal Neurological Institute, missing results indicate failed tractography.

The DRTT is the main efferent pathway from the cerebellum to the cerebral cortex and an important subdivision of the SCP in the context of deep brain stimulation-based neurosurgery. It originates in the dentate nucleus of the cerebellum, ascends through the brain stem where the majority of its constituent axons cross the midline at the superior cerebellar decussation (SCP) to synapse with the contra-lateral red nucleus (RN) in the midbrain (Coenen et al., 2020; Kwon et al., 2011; Mollink et al., 2016; Nowacki et al., 2018), see Fig. 19. From here the DRTT continues to the ventrolateral (VL) and ventromedial (VM) nuclei of the thalamus. Technically the DRTT terminates in the thalamus; however, for functional completeness we extend its trajectory to terminate in the primary motor cortex.

Whilst an ipsilateral component of the DRTT has been described in the literature, for the current purpose we have limited the reconstruction to the classical/contralateral DRTT. The left DRTT reconstruction failed in 4 datasets, while the right failed in 9 datasets. The overall DRTT wDSC showed a median of 0.498, minimum of 0.078 and Max.A.I.D. of 0.203. Additionally, we generated template DRTT bundles with 2,000 and 50,000 streamlines to illustrate the influence of the number streamline seeding attempts on tracking outcome and subsequent template-based reproducibility (supplementary Fig. 1).

##### 3.1.4.2. Inferior cerebellar peduncle (ICP).

The ICP consists mainly of afferent sensory fibers projecting from the spinal cord to the cerebellum. The ICPs are functionally involved in the maintenance of balance and posture via the integration of proprioceptive sensory and motor functions (Voogd, 2004). Microdissection studies reveal that it consists of four afferent bundles and 1 efferent bundle (Lingford-Hughes and Kalk, 2012), however here we reconstruct it as a single lateralized bundle using the dentate, fastigial and interposed nuclei of the cerebellum, and the ipsilateral medulla oblongata (Salamon et al., 2007; van Baarsen et al., 2016), see Fig. 19.

##### 3.1.4.3. Middle cerebellar peduncle (MCP).

The MCPs are large paired bundles that connect the brainstem to the cerebellum on both sides. They are often reconstructed in neuroimaging studies as a single commissural bundle connecting both cerebellar hemispheres (Leitner et al., 2015). The MCPs are thought to play a role in the modulation of skilled manual motor functions (Lingford-Hughes and Kalk, 2012) and have been shown to consist of three sub-fascicles (superior, inferior and deep) by microdissection studies (Jhaveri et al., 2018). We reconstructed the MCP as a whole for each side using the contra-lateral pons and cerebellar cortex. The resulting streamlines cross the midline in the pons, in line with its neuroanatomical definition. (Re et al., 2017; van Baarsen et al., 2016; Voogd, 2004), see Fig. 19.

### 3.2. HCP test‐retest data results

**Fig. 20.**
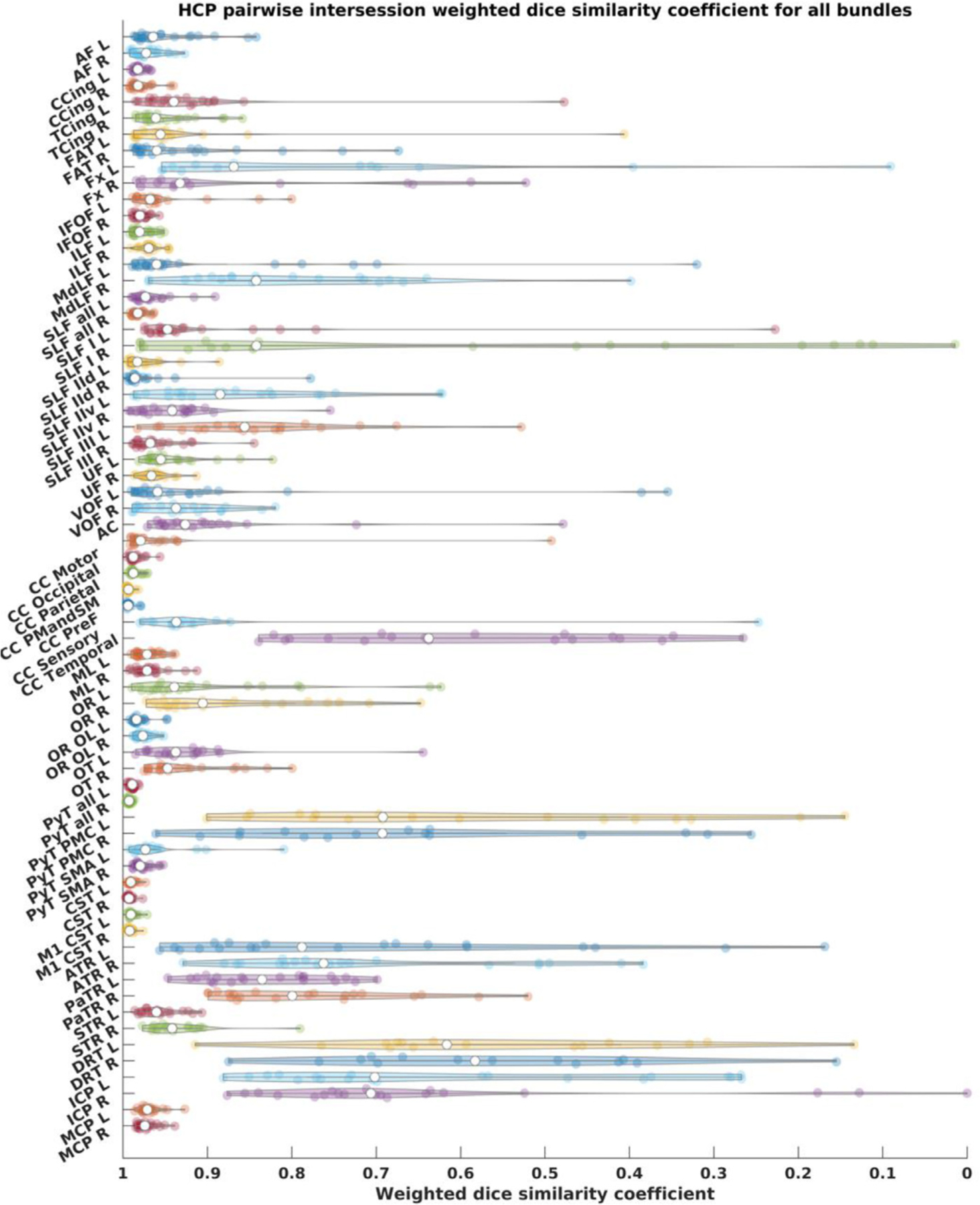
HCP tractograms pairwise wDSC scores depicted as violin plots. wDSC = weighted dice score, AF = arcuate fasciculus, CCing = cingulate cingulum, TCing = temporal cingulum, FAT = frontal aslant tract, Fx = fornix, IFOF = inferior fronto-occipital fasciculus, ILF = inferior longitudinal fasciculus, MdLF = middle longitudinal fasciculus, SLF = superior longitudinal fasciculus, SLF-IId = SLF-II dorsal division, SLF-IIv = SLF-II ventral division, UF = uncinate fasciculus, VOF = vertical occipital fasciculus, ML = medial lemniscus, OR = optic radiation, OR OL = optic radiation (using whole occipital lobe as cortical inclusion), OT = optic tract, PyT = pyramidal tract, CST = corticospinal tract, M1 = primary motor cortex, PyT = pyramidal tract, PMC = premotor cortex, SMA = supplementary motor area, ThR = thalamic radiation, Ant = anterior, Par = parietal, Sup = superior, Ant Comm = anterior commissure, CC = corpus callosum, PMC and SMA = premotor cortex and supplementary motor area, DRT = dentato-rubro-thalamic tract, ICP = inferior cerebellar peduncle, MCP = middle cerebellar peduncle, L = left, R = right.

In total we successfully reconstructed 2672 out of the 2720 attempted bundle reconstructions. The following bundles could not be reconstructed in all datasets: the DRTT (15 failures), premotor pyramidal tract (6 failures), optic tract (3 failures), fornix (19 failures), anterior commissure (2 failures), sensory CC (4 failures), and temporal CC (1 failure). Results of inter-session pair-wise similarity analysis per bundle for the different HCP test-retest subjects are shown as violin plots in Fig. 20.

Voxel-wise binary union maps per bundle show an expected pattern of maximum agreement in bundle cores and maximum variability around the periphery, supplementary Figs 2. and 3.

### 3.3. MASSIVE data results

**Fig. 21.**
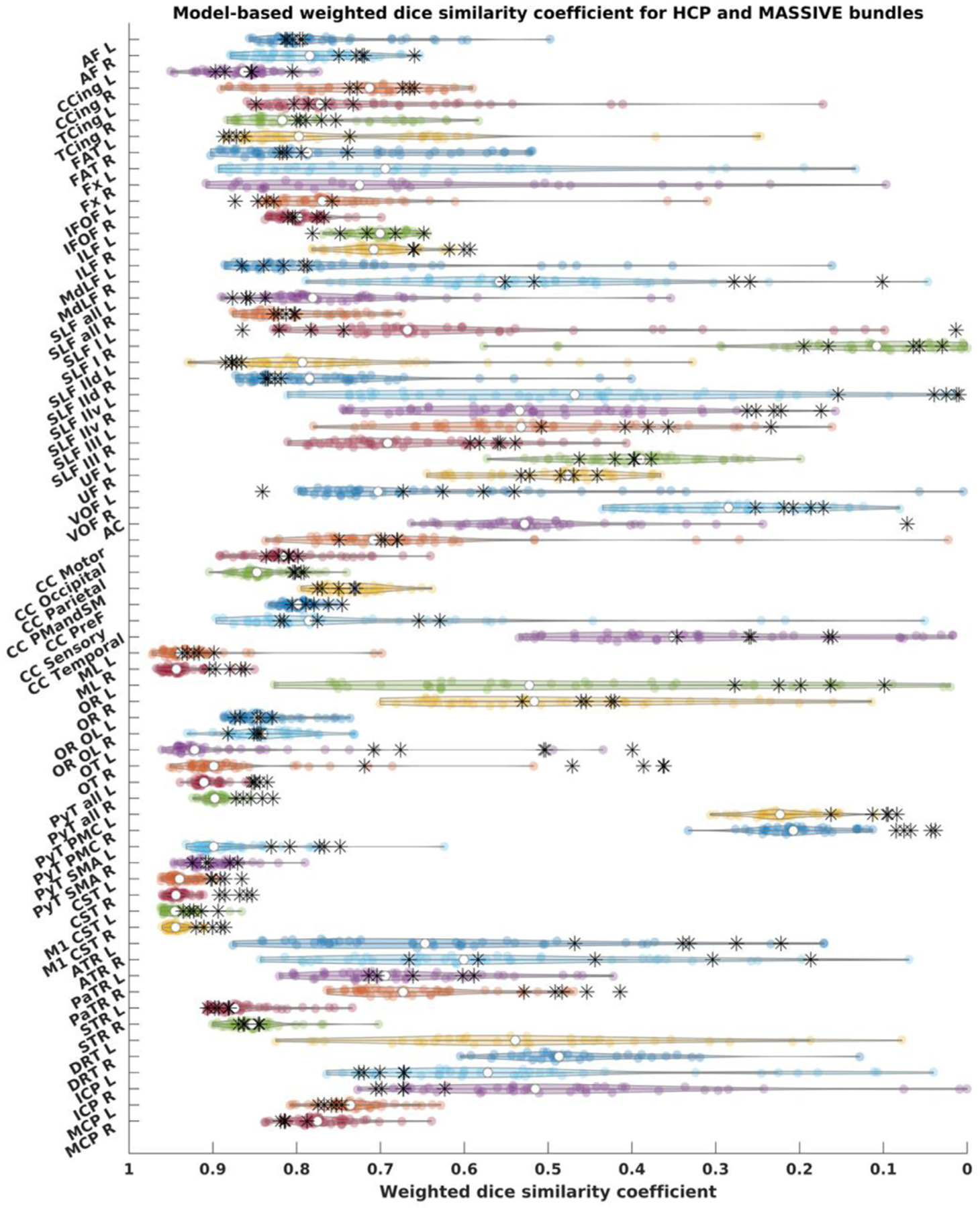
Weighted dice similarity coefficient scores for all bundles, HCP test-retest tractograms are depicted as violin plots and MASSIVE results are depicted as black asterisks., AF = arcuate fasciculus, CCing = cingulate cingulum, TCing = temporal cingulum, FAT = frontal aslant tract, Fx = fornix, IFOF = inferior fronto-occipital fasciculu, ILF = inferior longitudinal fasciculus, MdLF = middle longitudinal fasciculus, SLF = superior longitudinal fasciculus, SLF-IId = SLF-II dorsal division, SLF-IIv = SLF-II ventral division, UF = uncinate fasciculus, VOF = vertical occipital fasciculus, ML = medial lemniscus, OR = optic radiation, OR OL = optic radiation (using whole occipital lobe as cortical inclusion), OT = optic tract, PyT = pyramidal tract, CST = corticospinal tract, M1 = primary motor cortex, PyT = pyramidal tract, PMC = premotor cortex, SMA = supplementary motor area, ThR = thalamic radiation, Ant = anterior, Par = parietal, Sup = superior, Ant Comm = anterior commissure, CC = corpus callosum, PMC and SMA = premotor cortex and supplementary motor area, DRT = dentato-rubro-thalamic tract, ICP = inferior cerebellar peduncle, MCP = middle cerebellar peduncle, L = left, R = right.

Fig. 21 shows the wDSC scores derived from comparing the MASSIVE bundles to the HCP-template bundles superimposed on the results of the HCP test-retest dataset for reference. The single score agreement ICC was 0.917 (upper-bound = 0.944, lower-bound = 0.884, *p* < 0.05).

In total we successfully reconstructed 316 out of 340 bundles (68 for each of the 5 MASSIVE datasets). 63 out of the 68 bundles were generated for every dataset, while the anterior commissure was only generated in 1 dataset, and the DRTT and fornix were not generated in any dataset.

## 4. Discussion

This work aims to facilitate the use of advanced tractography methods within the clinical research community by providing both an educational reference and the open-source tractography pipeline FWT for researchers and clinicians wishing to apply CSD tractography to typical HARDI datasets. We demonstrate the variability of the reconstructed bundles within and between subjects using two test-retest datasets and offer solutions to improve tracking results for the least reproducible bundles.

### 4.1. Variability of virtual dissections

Our results demonstrate that inter-bundle variability was much greater than inter-subject variability for the same bundles i.e., there was a wide range of similarity in the extent to which different bundles were reconstructed irrespective of the dataset used. The AF and PyT for example were reconstructed in largely the same way in all datasets, whereas the fornix and DRTT showed high variability in density and spatial extent and were sometimes not reconstructed at all. In line with this, inter-subject agreement was highest for bundles with high streamline densities e.g., PyT, AF, CC and lowest for bundles with low streamline densities e.g., fornix, and DRTT.

Inter-session and intra-subject agreement were high for all bundles i.e., bundles were reconstructed largely to the same extent, (or failed to be reconstructed) in both sessions of the HCP test-retest dataset. In the test-retest data from a single-subject with varying *b-*values and shell schemes (MASSIVE) changing the *b-*value(s), number of gradient directions or shell scheme did not consistently alter bundle similarity to the model.

In agreement with other studies our results show considerable variability, influenced not only by data acquisition parameters such as scanner manufacturer, *b-*value, diffusion sampling scheme, reconstruction model (Schilling et al., 2021a), but also by the virtual dissection protocol itself (Schilling et al., 2021b). In the context of our study, despite using exactly the same WM dissection protocol for the HCP-template as for the individual datasets, none of the tracked bundles failed in the template data, while some failed in the individual datasets. This highlights the importance of data quality, as group-averaged data tends to have a higher signal-to-noise ratio (SNR) and increased blurriness in fine cortical structures due to inter-subject variability. These results demonstrate that even in a unified fully automated WM dissection protocol considerable inter-bundle and inter-subject variability remain. In contrast to previous work, we found that data acquisition parameters and sampling schemes had only a limited impact on bundle variability. This is likely due to the high number of volumes used per session, which would result in a higher SNR compared to the typical single-shell acquisition datasets used in clinical settings.

In agreement with other studies (Bonilha et al., 2015; Cousineau et al., 2017; Gu et al., 2017; Zhang et al., 2019), we found that larger bundles, particularly if they have both a larger volume and a higher streamline count, tend to be more similar/reproducible regardless of differences in scanning parameters or even normal inter-subject anatomical variations. It follows that automated procedures are most appropriate for fasciculi with these characteristics. For smaller, less dense or more narrow bundles such as the fornix, DRTT (and relatedly, the superior cerebellar peduncle) or specific subdivisions of larger bundles (e.g., SLF-I, temporal corpus callosum) we recommend using either data with sub-millimeter spatial resolution, bundle-specific manual dissection or using the provided template bundles in an automated RecoBundles (Garyfallidis et al., 2018) based workflow rather than the VOI-based approach. Of note, we also provide symmetrical versions of the HCP-template bundles along with aligned reference anatomical images. In line with the use of template bundles, FWT is compatible with RecoBundles (Garyfallidis et al., 2018) based pipelines that can overcome the need for structural parcellation, which can be particularly useful in clinical data to avoid false tractography results due to structural parcellation errors.

### 4.2. The benefits of using a CSD atlas in clinical studies

There are currently a number of diffusion-based tractography atlases and protocols available (Catani and Thiebaut de Schotten, 2008; Thiebaut de Schotten et al., 2011; Verhoeven et al., 2010; Wakana et al., 2004; Warrington et al., 2020; Wasserthal et al., 2018; Yendiki et al., 2011) some of which are based on HCP data (Hansen et al., 2020; Yeh et al., 2018). Here we aimed to primarily address the limitations of DTI based virtual dissection protocols, which predominate in clinical studies, either as part of comparative studies with healthy controls, or in a healthcare setting using data acquired in shorter scan times than are achievable in a research environment. We did not include formal comparisons to other HARDI/higher-order modelling-based atlases which precludes us from drawing conclusions about the relative benefits of FWT over other methods for virtual dissection in this context. It is our aim to complement other atlases based on detailed anatomical research and specialized acquisition schemes with a comprehensive description of CSD-based human WM anatomy which improves upon classical DTI representations in 3T data. Whilst the results presented here are based on high quality data in healthy subjects, we also tested our protocol on lower quality clinical data during the development phase of FWT, to ensure its translatability to clinical populations (see supplementary Fig. 9). A formal analysis relating to the application of FWT in patients with lesions is beyond the scope of this paper focusing on typical CSD anatomy and will be presented in a follow-up publication.

### 4.3. Technical considerations and limitations

This work relied on imaging data from only 20 individuals, however by using re-test scans yielding 40 datasets and the additional analysis using the MASSIVE dataset, our sample is sufficient to study test-retest reproducibility, as well as inter-subject variability. While our dissection protocol was literature-based and guided by several contemporary publications we did not include a direct comparison to any other atlas, nor did we apply formal specific criteria for assessing the validity of the definitions. Given the lack of consensus on anatomical definitions relating to WM fasciculi, it is possible that some readers will disagree with our interpretation, and that the FWT VOIs and reconstructions may need to be revised as the field evolves.

We attempted to keep rigid heuristic decisions (e.g., statistical thresholds) to a minimum especially those pertaining to streamlines filtering, where we utilized largely data-driven streamline filtering tools from Dipy (Garyfallidis et al., 2014) rather than relying on a more stringent selection of anatomical exclude VOIs. However, in order to cater to varying data quality, our choice of inclusion VOIs in some situations are influenced by considerations made for lower quality datasets than the ones utilized in this work. For example, for the AF we excluded any streamlines involving the precentral gyrus to avoid false streamlines tunneling through volume average voxels, which we have observed in lower quality single-shell data. Similarly, the AC is generated without the olfactory component, and we do not include subdivisions to the AF, CST, full posterior thalamic radiation, or superior cerebellar peduncles proper. We also do not include more esoteric bundles such as the superior-anterior fasciculus (David et al., 2019), temporo-insular fasciculus (Nachtergaele et al., 2019; Radwan et al., 2019), and inferior (auditory) thalamic radiation (Maffei et al., 2019a, 2019b), or those with a high chance of failure due to their geometry, such as the posterior commissure, or cranial nerves.

FWT tractography utilizes spherical-deconvolution informed filtering of tractograms (SIFT) (Smith et al., 2015, 2013)) based streamlines seeding and filtering for whole brain tractography, while further improvement can be expected if an optimized whole brain tractography method is utilized, e.g., global tractography (Christiaens et al., 2015) or particle-filtering tractography (Girard et al., 2014). FWT does not use a specific required number of output streamlines during bundle segmentation from whole-brain tractograms. However, this is necessary if the bundle specific approach is utilized. In this case the resulting bundles will be directly influenced by the required number of streamlines as this will influence the number of streamlines seeding attempts. This point is illustrated in supplementary Fig. 1, which shows that when the DRTT template bundles are generated with 2,000 and 50,000 required streamlines, the outcome from the 50,000 streamlines appears more complete as expected. Furthermore, when applied to the individual datasets, the bundle-specific approach with 20,000 required streamlines reconstructed more bundles successfully than the whole-brain tractography and bundle segmentation approach (73 vs. 67), and resulted in higher wDSC scores particularly when compared to the 50,000 streamlines DRTT template bundles. Tracking such challenging bundles may also benefit from anatomically-constrained tractography (Smith et al., 2012), which will be adopted in future versions of FWT.

The FWT pipeline is time-consuming, as FS recon-all alone requires at least 4–6 h and dMRI preprocessing can vary widely depending on acquisition parameters, and reconstruction method. E.g., whole brain tractography can take more than 4 h for a multi-shell multi-tissue CSD model with probabilistic tractography using second-order integration over distributions of fODFs (iFOD2) (Tournier et al., 2010) with fODF-driven dynamic seeding (Smith et al., 2015). Whilst this is offset by the flexibility of FWT allowing a more targeted approach using bundle-specific seeding and tractography in case of presurgical mapping, further development will focus on improving processing time. Additionally, future work will provide a clustering-based workflow independent of prior structural parcellation, the addition of more fiber bundles, as well as generating deterministic versions of all bundles. Finally, any justified and necessary changes to the inclusion/exclusion VOIs for any bundle can be easily implemented by changing the FWT workflows, which are openly provided, and a new atlas of bundles can be easily created by rerunning the template workflows on the HCP-group template or similar data.

## 5. Conclusion

The FWT pipeline can reconstruct 68 WM fasciculi and shows high inter- and intra-subject reproducibility. Dense bundles such as the PyT, AF and MCP yield the most reliable and least variable reconstructions. Higher resolution data or tract specific modification may be required for thinner bundles such as the fornix, DRTT, SLF I and premotor PyT. The FWT CSD atlas may be a useful reference and virtual dissection tool for applied neuroimaging students and clinical professionals wishing to use and understand the capabilities of CSD tractography.

## Supplementary Material

Tables

## Figures and Tables

**Table 1:** Diffusion weightings and total number of volumes.

Data\dMRI parameters		Number of volumes per shell	Diffusion weighting shell (*b-*values in s/mm^2^)	Total number of volumes
HCP test-retest data		18, 90, 90, 90	0, 1000, 2000, 3000	288
MASSIVE	1	60, 126, 251, 126	0, 500, 1000, 2000	563
datasets	2	60, 125, 251, 251	0, 2000, 3000, 4000	687
	3	30, 251	0, 1000	281
	4	30, 251	0, 2000	281
	5	30, 251	0, 3000	281

## Abbreviations

AC: anterior commissure
AC/PC: anterior commissure/posterior commissure
AF: arcuate fasciculus
AP: anterior-posterior
ATR: anterior thalamic radiation
BIDS: brain imaging data structure
CC: corpus callosum
CG/cing: cingulum
CSD: constrained spherical deconvolution
CST: cortico spinal tract
DL: dorso-lateral nucleus of the thalamus
DM: dorso-medial nucleus of the thalamus
dMRI: diffusion magnetic resonance imaging
DRTT: dentato-rubro-thalamic tract
DSC: dice similarity coefficient
DSI: diffusion spectral imaging
DTI: diffusion tensor imaging
FA: fractional anisotropy
FACT: fiber assignment by continuous tracking
FAT: frontal aslant tract
fODF: fiber orientation distribution function
FS: freeSurfer
FT: fiber tracking/tractography
FWT: fun with tracts
Fx: fornix
HARDI: high angular resolution diffusion imaging
HCP: human connectome project
ICC: intra-class correlation
ICP: inferior cerebellar peduncle
IFOF: inferior Fronto-occipital fasciculus
ILF: inferior longitudinal fasciculus
M1: primary motor cortex’
MALP-EM: multi-atlas label propagation with expectation maximization
MASSIVE: multiple acquisitions for standardization of structural imaging validation and evaluation
MCP: middle cerebellar peduncle
MdLF: middle longitudinal fasciculus
ML: medial lemniscus
MR: magnetic resonance
MRI: magnetic resonance imaging
MSBP: multiscale brain parcellator
NIST: neuroimaging and surgical technologies
OR: optic radiation
OT: optic tract
PaTR: parietal thalamic radiation
PD25: Parkinson’s disease 25 subjects histological atlas
PoTR: posterior thalamic radiation
PyT: pyramidal tract
ROIs: regions of interest
SLF: superior longitudinal fasciculus
SNR: signal-to-noise ratio
STR: superior thalamic radiation
SUIT: spatially unbiased atlas template of the cerebellum and brainstem
UF: uncinate fasciculus
UKBB: United Kingdom biobank
VOF: vertical occipital fasciculus
VOIs: volumes of interest
VPL: ventral postero-lateral
VPM: ventral postero-medial
wDSC: weighted dice similarity coefficient
WM: white matter

## References

[R1] Almairac F, Herbet G, Moritz-Gasser S, de Champfleur NM, Duffau H, 2015. The left inferior fronto-occipital fasciculus subserves language semantics: a multilevel lesion study. Brain Struct. Funct 220, 1983–1995. doi:10.1007/s00429-014-0773-1. 24744151

[R2] Andersson JLR, Skare S, Ashburner J, 2003a. How to correct susceptibility distortions in spin-echo echo-planar images: application to diffusion tensor imaging. Neuroimage 20, 870–888. doi:10.1016/S1053-8119(03)00336-7. 14568458

[R3] Andersson JLR, Skare S, Ashburner J, 2003b. How to correct susceptibility distortions in spin-echo echo-planar images: application to diffusion tensor imaging. Neuroimage 20, 870–888. doi:10.1016/S1053-8119(03)00336–7. 14568458

[R4] Andersson JLR, Sotiropoulos SN, 2016. An integrated approach to correction for off-resonance effects and subject movement in diffusion MR imaging. Neuroimage 125, 1063–1078. doi:10.1016/j.neuroimage.2015.10.019. 26481672 PMC4692656

[R5] Andersson JLR, Sotiropoulos SN, 2015. Non-parametric representation and prediction of single- and multi-shell diffusion-weighted MRI data using Gaussian processes. Neuroimage 122, 166–176. doi:10.1016/j.neuroimage.2015.07.067. 26236030 PMC4627362

[R6] Avants BB, Tustison NJ, Song G, Cook PA, Klein A, Gee JC, Gee C, 2011. A reproducible evaluation of ANTs similarity metric performance in brain image registration. Neuroimage 54, 2033–2044. doi:10.1016/j.neuroimage.2010.09.025.A. 20851191 PMC3065962

[R7] Bain JS, Yeatman JD, Schurr R, Rokem A, Mezer AA, 2019. Evaluating arcuate fasciculus laterality measurements across dataset and tractography pipelines. Hum. Brain Mapp 40, 3695–3711. doi:10.1002/hbm.24626. 31106944 PMC6679767

[R8] Barbeau EB, Descoteaux M, Petrides M, 2020. Dissociating the white matter tracts connecting the temporo-parietal cortical region with frontal cortex using diffusion tractography. Sci. Rep 10, 8186. doi:10.1038/s41598-020-64124-y. 32424290 PMC7235086

[R9] Basser PJ, Mattiello J, LeBihan D, 1994a. MR diffusion tensor spectroscopy and imaging. Biophys. J 66, 259–267. doi:10.1016/S0006-3495(94)80775-1. 8130344 PMC1275686

[R10] Basser PJ, Mattiello J, LeBihan D, 1994b. Estimation of the effective self-diffusion tensor from the NMR spin echo. J. Magn. Reson. B 103, 247–254. doi:10.1006/jmrb.1994.1037. 8019776

[R11] Bastiani M, Cottaar M, Fitzgibbon SP, Suri S, Alfaro-Almagro F, Sotiropoulos SN, Jbabdi S, Andersson JLR, 2019. Automated quality control for within and between studies diffusion MRI data using a non-parametric framework for movement and distortion correction. Neuroimage 184, 801–812. doi:10.1016/j.neuroimage.2018.09.073. 30267859 PMC6264528

[R12] Bayrak RG, Wang X, Schilling KG, Greer JM, Hansen CB, Blaber JA, Williams O, Beason-Held LL, Resnick SM, Rogers BP, Landman BA, 2020. TractEM: fast protocols for whole brain deterministic tractography-based white matter atlas doi:10.1101/651935.

[R13] Behrens TEJ, Johansen-Berg H, Woolrich MW, Smith SM, Wheeler-Kingshott CAM, Boulby PA, Barker GJ, Sillery EL, Sheehan K, Ciccarelli O, Thompson AJ, Brady JM, Matthews PM, 2003. Non-invasive mapping of connections between human thalamus and cortex using diffusion imaging. Nat. Neurosci 6, 750–757. doi:10.1038/nn1075. 12808459

[R14] Bernard F, Zemmoura I, Ter Minassian A, Lemée J-M, Menei P, 2019. Anatomical variability of the arcuate fasciculus: a systematical review. Surg. Radiol. Anat 41, 889–900. doi:10.1007/s00276-019-02244-5. 31028450

[R15] Bloy L, Ingalhalikar M, Eavani H, Schultz RT, Roberts TPL, Verma R, 2012. White matter atlas generation using HARDI based automated parcellation. Neuroimage 59, 4055–4063. doi:10.1016/j.neuroimage.2011.08.053. 21893205 PMC3272315

[R16] Bonilha L, Gleichgerrcht E, Fridriksson J, Rorden C, Breedlove JL, Nesland T, Paulus W, Helms G, Focke NK, 2015. Reproducibility of the structural brain connectome derived from diffusion tensor imaging. PLoS One 10, e0135247. doi:10.1371/journal.pone.0135247. 26332788 PMC4557836

[R17] Bore A, Rheault F, Theaud G, Théberge A, 2021. Scilpy [WWW Document] URL https://github.com/scilus/scilpy (accessed 8.3.21).

[R18] Bosch-Bouju C, Hyland BI, Parr-Brownlie LC, 2013. Motor thalamus integration of cortical, cerebellar and basal ganglia information: implications for normal and parkinsonian conditions. Front. Comput. Neurosci 7. doi:10.3389/fncom.2013.00163. PMC3822295 24273509

[R19] Bouyagoub S, Dowell NG, Gabel M, Cercignani M, 2020. Comparing multiband and singleband EPI in NODDI at 3 T: what are the implications for reproducibility and study sample sizes? Magn. Reson. Mater. Phys. Biol. Med doi:10.1007/s10334-020-00897-7. PMC8338814 33315165

[R20] Calamuneri A, Arrigo A, Mormina E, Milardi D, Cacciola A, Chillemi G, Marino S, Gaeta M, Quartarone A, 2018. White matter tissue quantification at low b-values within constrained spherical deconvolution framework. Front. Neurol 9. doi:10.3389/fneur.2018.00716, 716–716. 30210438 PMC6122130

[R21] Catani M, 2006. Diffusion tensor magnetic resonance imaging tractography in cognitive disorders. Curr. Opin. Neurol 19, 599–606. doi:10.1097/01.wco.0000247610.44106.3f. 17102700

[R22] Catani M, Howard RJ, Pajevic S, Jones DK, 2002. Virtual in vivo interactive dissection of white matter fasciculi in the human brain. Neuroimage 17, 77–94. doi:10.1006/nimg.2002.1136. 12482069

[R23] Catani M, Thiebaut de Schotten M, 2008. A diffusion tensor imaging tractography atlas for virtual in vivo dissections. Cortex 44, 1105–1132. doi:10.1016/j.cortex.2008.05.004, Special Issue on “Brain Hodology - Revisiting disconnection approaches to disorders of cognitive function”. 18619589

[R24] Çavdar S, Aydın AE, Algın O, Aydın S, 2021. The complex structure of the anterior white commissure of the human brain: fiber dissection and tractography study. World Neurosurg 147, e111–e117. doi:10.1016/j.wneu.2020.11.157. 33290898

[R25] Caverzasi E, Hervey-Jumper SL, Jordan KM, Lobach IV, Li J, Panara V, Racine CA, Sankaranarayanan V, Amirbekian B, Papinutto N, Berger MS, Henry RG, 2016. Identifying preoperative language tracts and predicting postoperative functional recovery using HARDI q-ball fiber tractography in patients with gliomas. J. Neurosurg 125, 33–45. doi:10.3171/2015.6.JNS142203. 26654181

[R26] Chamberland M, Tax CMW, Jones DK, 2018. Meyer’s loop tractography for image-guided surgery depends on imaging protocol and hardware. NeuroImage Clin 20, 458–465. doi:10.1016/j.nicl.2018.08.021. 30128284 PMC6096050

[R27] Chen Z, Tie Y, Olubiyi O, Rigolo L, Mehrtash A, Norton I, Pasternak O, Rathi Y, Golby AJ, O’Donnell LJ, 2015. Reconstruction of the arcuate fasciculus for surgical planning in the setting of peritumoral edema using two-tensor unscented Kalman filter tractography. NeuroImage Clin 7, 815–822. doi:10.1016/j.nicl.2015.03.009. 26082890 PMC4459040

[R28] Chenot Q, Tzourio-Mazoyer N, Rheault F, Descoteaux M, Crivello F, Zago L, Mellet E, Jobard G, Joliot M, Mazoyer B, Petit L, 2019. A population-based atlas of the human pyramidal tract in 410 healthy participants. Brain Struct. Funct 224, 599–612. doi:10.1007/s00429-018-1798-7. 30460551

[R29] Choi H, Kubicki M, Whitford T, Alvarado JL, Terry DP, Niznikiewicz M, Mc-Carley RW, Kwon JS, Shenton ME, 2011. Diffusion tensor imaging of anterior commissural fibers in patients with schizophrenia. Schizophr. Res 130, 78–85. doi:10.1016/j.schres.2011.04.016. 21561738 PMC3745276

[R30] Christiaens D, Reisert M, Dhollander T, Sunaert S, Suetens P, Maes F, 2015. Global tractography of multi-shell diffusion-weighted imaging data using a multi-tissue model. Neuroimage 123, 89–101. doi:10.1016/j.neuroimage.2015.08.008. 26272729

[R31] Christiansen K, Metzler-Baddeley C, Parker GD, Muhlert N, Jones DK, Aggleton JP, Vann SD, 2017. Topographic separation of fornical fibers associated with the anterior and posterior hippocampus in the human brain: an MRI-diffusion study. Brain Behav 7, e00604. doi:10.1002/brb3.604. 28127522 PMC5256187

[R32] Coenen VA, Sajonz B, Prokop T, Reisert M, Piroth T, Urbach H, Jenkner C, Reinacher PC, 2020. The dentato-rubro-thalamic tract as the potential common deep brain stimulation target for tremor of various origin: an observational case series. Acta Neurochir 162, 1053–1066. doi:10.1007/s00701-020-04248-2, (Wien). 31997069 PMC7156360

[R33] Cordero-Grande L, Christiaens D, Hutter J, Price AN, Hajnal JV, 2019. Complex diffusion-weighted image estimation via matrix recovery under general noise models. Neuroimage 200, 391–404. doi:10.1016/j.neuroimage.2019.06.039. 31226495 PMC6711461

[R34] Cousineau M, Jodoin P-M, Garyfallidis E, Côté M-A, Morency FC, Rozanski V, Grand’Maison M, Bedell BJ, Descoteaux M, 2017. A test-retest study on Parkinson’s PPMI dataset yields statistically significant white matter fascicles. NeuroImage Clin 16, 222–233. doi:10.1016/j.nicl.2017.07.020. 28794981 PMC5547250

[R35] David S, Heemskerk AM, Corrivetti F, Thiebaut de Schotten M, Sarubbo S, Corsini F, De Benedictis A, Petit L, Viergever MA, Jones DK, Mandonnet E, Axer H, Evans J, Paus T, Leemans A, 2019. The superoanterior fasciculus (SAF): a novel white matter pathway in the human brain? Front. Neuroanat doi:10.3389/fnana.2019.00024, 0. PMC6412356 30890921

[R36] de Schotten MT, Dell’Acqua F, Forkel SJ, Simmons A, Vergani F, Murphy DGM, Catani M, 2011. A lateralized brain network for visuospatial attention. Nat. Neurosci 14, 1245–1246. doi:10.1038/nn.2905. 21926985

[R37] De Witt Hamer PC, Moritz-Gasser S, Gatignol P, Duffau H, 2010. Is the human left middle longitudinal fascicle essential for language? A brain electrostimulation study. Hum. Brain Mapp 32, 962–973. doi:10.1002/hbm.21082. 20578169 PMC6870476

[R38] Dick AS, Garic D, Graziano P, Tremblay P, 2019. The frontal aslant tract (FAT) and its role in speech, language and executive function. Cortex J. Devoted Study Nerv. Syst. Behav 111, 148–163. doi:10.1016/j.cortex.2018.10.015. PMC6461388 30481666

[R39] Dick AS, Tremblay P, 2012. Beyond the arcuate fasciculus: consensus and controversy in the connectional anatomy of language. Brain 135, 3529–3550. doi:10.1093/brain/aws222. 23107648

[R40] Diedrichsen J, 2006. A spatially unbiased atlas template of the human cerebellum. Neuroimage 33, 127–138. doi:10.1016/j.neuroimage.2006.05.056. 16904911

[R41] Diedrichsen J, Balsters JH, Flavell J, Cussans E, Ramnani N, 2009. A probabilistic MR atlas of the human cerebellum. NeuroImage 46, 39–46. doi:10.1016/j.neuroimage.2009.01.045. 19457380

[R42] Diedrichsen J, Maderwald S, Küper M, Thürling M, Rabe K, Gizewski ER, Ladd ME, Timmann D, 2011. Imaging the deep cerebellar nuclei: a probabilistic atlas and normalization procedure. Neuroimage 54, 1786–1794. doi:10.1016/j.neuroimage.2010.10.035. 20965257

[R43] Diedrichsen J, Zotow E, 2015. Surface-based display of volume-averaged cerebellar imaging data. PLoS One 10, e0133402. doi:10.1371/journal.pone.0133402. 26230510 PMC4521932

[R44] Duan F, Zhao T, He Y, Shu N, 2015. Test–retest reliability of diffusion measures in cerebral white matter: a multiband diffusion MRI study. J. Magn. Reson. Imaging 42, 1106–1116. doi:10.1002/jmri.24859. 25652348

[R45] Fabri M, Pierpaoli C, Barbaresi P, Polonara G, 2014. Functional topography of the corpus callosum investigated by DTI and fMRI. World J. Radiol 6, 895–906. doi:10.4329/wjr.v6.i12.895. 25550994 PMC4278150

[R46] Farquharson S, Tournier J-D, Calamante F, Fabinyi G, Schneider-Kolsky M, Jackson GD, Connelly A, 2013. White matter fiber tractography: why we need to move beyond DTI. J. Neurosurg 118, 1367–1377. doi:10.3171/2013.2.JNS121294. 23540269

[R47] Fernández-Miranda JC, Wang Y, Pathak S, Stefaneau L, Verstynen T, Yeh FC, 2015. Asymmetry, connectivity, and segmentation of the arcuate fascicle in the human brain. Brain Struct. Funct 220, 1665–1680. doi:10.1007/s00429-014-0751-7. 24633827

[R48] Fischl B, 2012. FreeSurfer. NeuroImage 62, 774–781. doi:10.1016/j.neuroimage.2012.01.021. 22248573 PMC3685476

[R49] Forkel SJ, Thiebaut de Schotten M, Kawadler JM, Dell’Acqua F, Danek A, Catani M, 2014. The anatomy of fronto-occipital connections from early blunt dissections to contemporary tractography. Cortex 56, 73–84. doi:10.1016/j.cortex.2012.09.005, The clinical neuroanatomy of the occipital lobes. 23137651

[R50] Froeling M, Tax CMW, Vos SB, Luijten PR, Leemans A, 2017. MASSIVE” brain dataset: multiple acquisitions for standardization of structural imaging validation and evaluation. Magn. Reson. Med 77, 1797–1809. doi:10.1002/mrm.26259. 27173617

[R51] Garyfallidis E, Brett M, Amirbekian B, Rokem A, Van Der Walt S, Descoteaux M, Nimmo-Smith I, 2014. Dipy, a library for the analysis of diffusion MRI data. Front. Neuroinform 8. doi:10.3389/fninf.2014.00008. PMC3931231 24600385

[R52] Garyfallidis E, Brett M, Correia MM, Williams GB, Nimmo-Smith I, 2012. QuickBundles, a method for tractography simplification. Front. Neurosci 6. doi:10.3389/fnins.2012.00175. PMC3518823 23248578

[R53] Garyfallidis E, Côté M-A, Rheault F, Sidhu J, Hau J, Petit L, Fortin D, Cunanne S, Descoteaux M, 2018. Recognition of white matter bundles using local and global streamline-based registration and clustering. Neuroimage Segm. Brain 170, 283–295. doi:10.1016/j.neuroimage.2017.07.015. 28712994

[R54] Girard G, Whittingstall K, Deriche R, Descoteaux M, 2014. Towards quantitative connectivity analysis: reducing tractography biases. Neuroimage 98, 266–278. doi:10.1016/j.neuroimage.2014.04.074. 24816531

[R55] Glasser MF, Sotiropoulos SN, Wilson JA, Coalson TS, Fischl B, Andersson JL, Xu J, Jbabdi S, Webster M, Polimeni JR, Van Essen DC, Jenkinson M, 2013. The minimal preprocessing pipelines for the human connectome project. Neuroimage 80, 105–124. doi:10.1016/j.neuroimage.2013.04.127. 23668970 PMC3720813

[R56] Goga C, Türe U, 2015. The anatomy of Meyer’s loop revisited: changing the anatomical paradigm of the temporal loop based on evidence from fiber microdissection. J. Neurosurg 122, 1253–1262. doi:10.3171/2014.12.JNS14281. 25635481

[R57] Gorgolewski KJ, Auer T, Calhoun VD, Craddock RC, Das S, Duff EP, Flandin G, Ghosh SS, Glatard T, Halchenko YO, Handwerker DA, Hanke M, Keator D, Li X, Michael Z, Maumet C, Nichols BN, Nichols TE, Pellman J, Poline J-BB, Rokem A, Schaefer G, Sochat V, Triplett W, Turner JA, Varoquaux G, Poldrack RA, 2016. The brain imaging data structure, a format for organizing and describing outputs of neuroimaging experiments. Sci. Data 3, 1–9. doi:10.1038/sdata.2016.44. PMC4978148 27326542

[R58] Gu X, Eklund A, Knutsson H, Schultz T, Özarslan E, Hotz I, 2017. Repeated tractography of a single subject: how high is the variance? In: Modeling, Analysis, and Visualization of Anisotropy, Mathematics and Visualization Springer International Publishing, Cham, pp. 331–354. doi:10.1007/978-3-319-61358-1_14.

[R59] Hansen CB, Yang Q, Lyu I, Rheault F, Kerley C, Chandio BQ, Fadnavis S, Williams O, Shafer AT, Resnick SM, Zald DH, Cutting L, Taylor WD, Boyd B, Garyfallidis E, Anderson AW, Descoteaux M, Landman BA, Schilling KG, 2020. Pandora: 4-D white matter bundle population-based atlases derived from diffusion MRI fiber tractography. bioRxiv 06 (12), 148999. doi:10.1101/2020.06.12.148999, 2020. PMC8124084 33196967

[R60] Hau J, Sarubbo S, Houde JC, Corsini F, Girard G, Deledalle C, Crivello F, Zago L, Mellet E, Jobard G, Joliot M, Mazoyer B, Tzourio-Mazoyer N, Descoteaux M, Petit L, 2017. Revisiting the human uncinate fasciculus, its subcomponents and asymmetries with stem-based tractography and microdissection validation. Brain Struct. Funct 222, 1645–1662. doi:10.1007/s00429-016-1298-6. 27581617

[R61] Heilbronner SR, Haber SN, 2014. Frontal cortical and subcortical projections provide a basis for segmenting the cingulum bundle: implications for neuroimaging and psychiatric disorders. J. Neurosci 34, 10041–10054. 25057206 10.1523/JNEUROSCI.5459-13.2014 PMC4107396

[R62] Herbet G, Zemmoura I, Duffau H, 2018. Functional anatomy of the inferior longitudinal fasciculus: from historical reports to current hypotheses. Front. Neuroanat 12. doi:10.3389/fnana.2018.00077. PMC6156142 30283306

[R63] Hofer S, Karaus A, Frahm J, 2010. Reconstruction and dissection of the entire human visual pathway using diffusion tensor MRI. Front. Neuroanat 4. doi:10.3389/fnana.2010.00015. PMC2859811 20428499

[R64] Hong X, Zheng L, Rajan A, Ding M, 2019. Role of superior longitudinal fasciculus in visual spatial attention. J. Vis 19. doi:10.1167/19.10.320, 320–320.

[R65] Jang SH, Seo JP, 2015. Differences of the medial lemniscus and spinothalamic tract according to the cortical termination areas: a diffusion tensor tractography study. Somatosens. Mot. Res 32, 67–71. doi:10.3109/08990220.2014.966899. 25365478

[R66] Jenkinson M, Bannister P, Brady M, Smith S, 2002. Improved optimization for the robust and accurate linear registration and motion correction of brain images. Neuroimage 17, 825–841. doi:10.1016/s1053-8119(02)91132-8. 12377157

[R67] Jenkinson M, Beckmann CF, Behrens TEJ, Woolrich MW, Smith SM, 2012. FSL. Neuroimage 62, 782–790. doi:10.1016/j.neuroimage.2011.09.015. 21979382

[R68] Jeong JW, Asano E, Yeh FC, Chugani DC, Chugani HT, 2013. Independent component analysis tractography combined with a ball-stick model to isolate intravoxel crossing fibers of the corticospinal tracts in clinical diffusion MRI. Magn. Reson. Med 70, 441–453. doi:10.1002/mrm.24487. 23001816 PMC3657599

[R69] Jeurissen B, Tournier J-DD, Dhollander T, Connelly A, Sijbers J, 2014. Multi-tissue constrained spherical deconvolution for improved analysis of multi-shell diffusion MRI data. Neuroimage 103, 411–426. doi:10.1016/j.neuroimage.2014.07.061. 25109526

[R70] Jhaveri MD, Salzman KL, Ross JS, Moore KR, Osborn AG, Ho CY, Jhaveri MD, Salzman KL, Ross JS, Moore KR, Osborn AG, Ho CY, 2018. Middle cerebellar peduncle lesion(s). In: Expertddx: Brain and Spine Elsevier, pp. 518–521. doi:10.1016/B978-0-323-44308-1.50200-2 ExpertDDx.

[R71] Jitsuishi T, Hirono S, Yamamoto T, Kitajo K, Iwadate Y, Yamaguchi A, 2020. White matter dissection and structural connectivity of the human vertical occipital fasciculus to link vision-associated brain cortex. Sci. Rep 10, 820. doi:10.1038/s41598-020-57837-7. 31965011 PMC6972933

[R72] Jones DK, Christiansen KF, Chapman RJ, Aggleton JP, 2013. Distinct subdivisions of the cingulum bundle revealed by diffusion MRI fibre tracking: implications for neuropsychological investigations. Neuropsychologia 51, 67–78. doi:10.1016/j.neuropsychologia.2012.11.018. 23178227 PMC3611599

[R73] Kakou M, Kouakou F, Nâ TM dri Oka D, Mbende AS, Peltier J, Velut S, 2017. Microanatomy of thalamic radiations. Int. J. Hum. Anat 1, 28–37. doi:10.14302/issn.2577-2279.ijha-17-1719.

[R74] Kalyvas A, Koutsarnakis C, Komaitis S, Karavasilis E, Christidi F, Skandalakis GP, Liouta E, Papakonstantinou O, Kelekis N, Duffau H, Stranjalis G, 2020. Mapping the human middle longitudinal fasciculus through a focused anatomo-imaging study: shifting the paradigm of its segmentation and connectivity pattern. Brain Struct. Funct 225, 85–119. doi:10.1007/s00429-019-01987-6. 31773331

[R75] Kikinis Z, Fitzsimmons J, Dunn C, Vu M-A, Makris N, Bouix S, Goldstein JM, Mesholam-Gately RI, Petryshen T, del Re EC, Wojcik J, Seidman LJ, Kubicki M, 2015. Anterior commissural white matter fiber abnormalities in first-episode psychosis: a tractography study. Schizophr. Res 162, 29–34. doi:10.1016/j.schres.2015.01.037. 25667192 PMC4339098

[R76] Kreilkamp BAK, Lisanti L, Glenn GR, Wieshmann UC, Das K, Marson AG, Keller SS, 2019. Comparison of manual and automated fiber quantification tractography in patients with temporal lobe epilepsy. NeuroImage Clin 24, 102024. doi:10.1016/j.nicl.2019.102024. 31670154 PMC6831895

[R77] Kurki TJI, Laalo JP, Oksaranta OM, 2013. Diffusion tensor tractography of the uncinate fasciculus: Pitfalls in quantitative analysis due to traumatic volume changes. J. Magn. Reson. Imaging 38, 46–53. doi:10.1002/jmri.23901. 23733545

[R78] Kwon HG, Hong JH, Hong CP, Lee DH, Ahn SH, Jang SH, 2011. Dentatorubrothalamic tract in human brain: diffusion tensor tractography study. Neuroradiology 53, 787–791. doi:10.1007/s00234-011-0878-7. 21547376

[R79] La Corte E, Eldahaby D, Greco E, Aquino D, Bertolini G, Levi V, Ottenhausen M, Demichelis G, Romito LM, Acerbi F, Broggi M, Schiariti MP, Ferroli P, Bruzzone MG, Serrao G, 2021. The frontal aslant tract: a systematic review for neurosurgical applications. Front. Neurol 12. doi:10.3389/fneur.2021.641586. PMC7959833 33732210

[R80] Larkman DJ, Hajnal JV, Herlihy AH, Coutts GA, Young IR, Ehnholm G, 2001. Use of multicoil arrays for separation of signal from multiple slices simultaneously excited. J. Magn. Reson. Imaging JMRI 13, 313–317. doi:10.1002/1522-2586(200102)13:2<313::aid-jmri1045>3.0.co;2-w. 11169840

[R81] Latini F, Mårtensson J, Larsson E-M, Fredrikson M, Åhs F, Hjortberg M, Aldskogius H, Ryttlefors M, 2017. Segmentation of the inferior longitudinal fasciculus in the human brain: A white matter dissection and diffusion tensor tractography study. Brain Res 1675, 102–115. doi:10.1016/j.brainres.2017.09.005. 28899757

[R82] Leitner Y, Travis KE, Ben-Shachar M, Yeom KW, Feldman HM, 2015. Tract profiles of the cerebellar white matter pathways in children and adolescents. Cerebellum 14, 613–623. doi:10.1007/s12311-015-0652-1. 25648754 PMC4524802

[R83] Liacu D, Idy-Peretti I, Ducreux D, Bouilleret V, De Marco G, 2012. Diffusion tensor imaging tractography parameters of limbic system bundles in temporal lobe epilepsy patients. J. Magn. Reson. Imaging 36, 561–568. doi:10.1002/jmri.23678. 22552939

[R84] Lim JC, Phal PM, Desmond PM, Nichols AD, Kokkinos C, Danesh-Meyer HV, Kaye AH, Moffat BA, 2015. Probabilistic MRI tractography of the optic radiation using constrained spherical deconvolution: a feasibility study. PLoS One 10. doi:10.1371/journal.pone.0118948, e0118948–e0118948. 25742640 PMC4351098

[R85] Lingford-Hughes A, Kalk N, Wright P, Stern J„ Phelan M, 2012. 2 - Clinical neuroanatomy. In: Core Psychiatry Saunders WB, Oxford, pp. 13–34. doi:10.1016/B978-0-7020-3397-1.00002-1.

[R86] Lustig M, Donoho D, Pauly JM, 2007. Sparse MRI: the application of compressed sensing for rapid MR imaging. Magn. Reson. Med 58, 1182–1195. doi:10.1002/mrm.21391. 17969013

[R87] Lustig M, Donoho DLDDL, Santos JMJMM, Pauly JMJMM, 2008. Compressed sensing MRI. IEEE Signal Process. Mag 25, 72–82. doi:10.1109/Tit.2006.871582.

[R88] Lyness RC, Alvarez I, Sereno MI, MacSweeney M, 2014. Microstructural differences in the thalamus and thalamic radiations in the congenitally deaf. Neuroimage 100, 347–357. doi:10.1016/j.neuroimage.2014.05.077. 24907483 PMC4148523

[R89] Madhavan KM, McQueeny T, Howe SR, Shear P, Szaflarski J, 2014. Superior longitudinal fasciculus and language functioning in healthy aging. Brain Res 1562, 11–22. doi:10.1016/j.brainres.2014.03.012. 24680744 PMC4049076

[R90] Maffei C, Lee C, Planich M, Ramprasad M, Ravi N, Trainor D, Urban Z, Kim M, Jones RJ, Henin A, Hofmann SG, Pizzagalli DA, Auerbach RP, Gabrieli JDE, Whitfield-Gabrieli S, Greve DN, Haber SN, Yendiki A, 2021. Using diffusion MRI data acquired with ultra-high gradient strength to improve tractography in routine-quality data. NeuroImage 245, 118706. doi:10.1016/j.neuroimage.2021.118706. 34780916 PMC8835483

[R91] Maffei C, Sarubbo S, Jovicich J, 2019a. A Missing connection: a review of the macrostructural anatomy and tractography of the acoustic radiation. Front. Neuroanat 0. doi:10.3389/fnana.2019.00027. PMC6416820 30899216

[R92] Maffei C, Sarubbo S, Jovicich J, 2019b. Diffusion-based tractography atlas of the human acoustic radiation. Sci. Rep 9, 4046. doi:10.1038/s41598-019-40666-8. 30858451 PMC6411970

[R93] Maier-Hein KH, Neher PF, Houde JC, Côté MA, Garyfallidis E, Zhong J, Chamberland M, Yeh FC, Lin YC, Ji Q, Reddick WE, Glass JO, Chen DQ, Feng Y, Gao C, Wu Y, Ma J, He R, Li Q, Westin CF, Deslauriers-Gauthier S, González JOO, Paquette M, St-Jean S, Girard G, Rheault F, Sidhu J, Tax CMW, Guo F, Mesri HY, Dávid S, Froeling M, Heemskerk AM, Leemans A, Boré A, Pinsard B, Bedetti C, Desrosiers M, Brambati S, Doyon J, Sarica A, Vasta R, Cerasa A, Quattrone A, Yeatman J, Khan AR, Hodges W, Alexander S, Romascano D, Barakovic M, Auría A, Esteban O, Lemkaddem A, Thiran JP, Cetingul HE, Odry BL, Mailhe B, Nadar MS, Pizzagalli F, Prasad G, Villalon-Reina JE, Galvis J, Thompson PM, Requejo FDS, Laguna PL, Lacerda LM, Barrett R, Dell’Acqua F, Catani M, Petit L, Caruyer E, Daducci A, Dyrby TB, Holland-Letz T, Hilgetag CC, Stieltjes B, Descoteaux M, 2017. The challenge of mapping the human connectome based on diffusion tractography. Nat. Commun 8, 1349. doi:10.1038/s41467-017-01285-x. 29116093 PMC5677006

[R94] Makris N, Papadimitriou GM, Kaiser JR, Sorg S, Kennedy DN, Pandya DN, 2009. Delineation of the middle longitudinal fascicle in humans: a quantitative, in vivo, DT-MRI study. Cereb. Cortex 19, 777–785. doi:10.1093/cercor/bhn124, N. Y. N 1991. 18669591 PMC2651473

[R95] Makris N, Zhu A, Papadimitriou GM, Mouradian P, Ng I, Scaccianoce E, Baselli G, Baglio F, Shenton ME, Rathi Y, Dickerson B, Yeterian E, Kubicki M, 2017. Mapping temporo-parietal and temporo-occipital cortico-cortical connections of the human middle longitudinal fascicle in subject-specific, probabilistic, and stereotaxic Talairach spaces. Brain Imaging Behav 11, 1258–1277. doi:10.1007/s11682-016-9589-3. 27714552 PMC5382125

[R96] Maldonado IL, de Champfleur NM, Velut S, Destrieux C, Zemmoura I, Duffau H, 2013. Evidence of a middle longitudinal fasciculus in the human brain from fiber dissection. J. Anat 223, 38–45. doi:10.1111/joa.12055. 23621438 PMC3798102

[R97] Martínez-Heras E, Varriano F, Prčkovska V, Laredo C, Andorrà M, Martínez-Lapiscina EH, Calvo A, Lampert E, Villoslada P, Saiz A, Prats-Galino A, Llufriu S, 2015. Improved framework for tractography reconstruction of the optic radiation. PLoS One 10. doi:10.1371/journal.pone.0137064, e0137064–e0137064. 26376179 PMC4573981

[R98] Meesters S, Ossenblok P, Wagner L, Schijns O, Boon P, Florack L, Vilanova A, Duits R, 2017. Stability metrics for optic radiation tractography: towards damage prediction after resective surgery. J. Neurosci. Methods 288, 34–44. doi:10.1016/j.jneumeth.2017.05.029. 28648721 PMC5538260

[R99] Mehra D, Moshirfar M, 2021. Neuroanatomy, Optic Tract. StatPearls StatPearls Publishing, Treasure Island (FL) 31751030

[R100] Menjot de Champfleur N, Lima Maldonado I, Moritz-Gasser S, Machi P, Le Bars E, Bonafé A, Duffau H, 2013. Middle longitudinal fasciculus delineation within language pathways: a diffusion tensor imaging study in human. Eur. J. Radiol 82, 151–157. doi:10.1016/j.ejrad.2012.05.034, Special Section: Imaging of the Peripheral Nervous System. 23084876

[R101] Metzler-Baddeley C, Jones DK, Steventon J, Westacott L, Aggleton JP, O’Sullivan MJ, 2012. Cingulum microstructure predicts cognitive control in older age and mild cognitive impairment. J. Neurosci 32, 17612–17619. 23223284 10.1523/JNEUROSCI.3299-12.2012 PMC6621654

[R102] Meynert T, 1888. Psychiatrie clinique des maladies du cerveau antérieur. Psychiatr. Clin. Mal. Cerveau Antér 294–294.

[R103] Miller KL, Alfaro-Almagro F, Bangerter NK, Thomas DL, Yacoub E, Xu J, Bartsch AJ, Jbabdi S, Sotiropoulos SN, Andersson JL, Griffanti L, Douaud G, Okell TW, Weale P, Dragonu I, Garratt S, Hudson S, Collins R, Jenkinson M, Matthews PM, Smith SM, 2016. Multimodal population brain imaging in the UK Biobank prospective epidemiological study. Nat. Neurosci 19, 1523–1536. doi:10.1038/nn.4393. 27643430 PMC5086094

[R104] Moeller S, Yacoub E, Olman CA, Auerbach E, Strupp J, Harel N, Uğurbil K, 2010. Multiband multislice GE-EPI at 7 tesla, with 16-fold acceleration using partial parallel imaging with application to high spatial and temporal whole-brain fMRI. Magn. Reson. Med 63, 1144–1153. doi:10.1002/mrm.22361. 20432285 PMC2906244

[R105] Mollink J, van Baarsen KM, Dederen PJWC, Foxley S, Miller KL, Jbabdi S, Slump CH, Grotenhuis JA, Kleinnijenhuis M, van Cappellen van Walsum AM, 2016. dentato-rubro-thalamic tract localization with postmortem MR diffusion tractography compared to histological 3D reconstruction. Brain Struct. Funct 221, 3487–3501. doi:10.1007/s00429-015-1115-7. 26438333 PMC5009171

[R106] Mori S, Crain BJ, Chacko VP, van Zijl PC, 1999. Three-dimensional tracking of axonal projections in the brain by magnetic resonance imaging. Ann. Neurol 45, 265–269. doi:10.1002/1531-8249(199902)45:2<265::aid-ana21gt;3.0.co;2-3. 9989633

[R107] Mori S, Oishi K, Faria AV, 2009. White matter atlases based on diffusion tensor imaging. Curr. Opin. Neurol 22, 362–369. doi:10.1097/WCO.0b013e32832d954b. 19571751 PMC2883814

[R108] Mori S, Oishi K, Jiang H, Jiang L, Li X, Akhter K, Hua K, Faria AV, Mahmood A, Woods R, Toga AW, Pike GB, Neto PR, Evans A, Zhang J, Huang H, Miller MI, van Zijl P, Mazziotta J, 2008. Stereotaxic white matter atlas based on diffusion tensor imaging in an ICBM template. Neuroimage 40, 570–582. doi:10.1016/j.neuroimage.2007.12.035. 18255316 PMC2478641

[R109] Mori S, Tournier JD, 2014. Chapter 8 - moving beyond dti: high angular resolution diffusion imaging (HARDI). In: Introduction to Diffusion Tensor Imaging Academic Press, San Diego, pp. 65–78. doi:10.1016/B978-0-12-398398-5.00008-4.

[R110] Nachtergaele P, Radwan A, Swinnen S, Decramer T, Uytterhoeven M, Sunaert S, van Loon J, Theys T, 2019. The temporoinsular projection system: an anatomical study. J. Neurosurg 22, 1–9. doi:10.3171/2018.11.JNS18679. 30797196

[R111] Nazem-Zadeh M-R, Saksena S, Babajani-Fermi A, Jiang Q, Soltanian-Zadeh H, Rosenblum M, Mikkelsen T, Jain R, 2012. Segmentation of corpus callosum using diffusion tensor imaging: validation in patients with glioblastoma. BMC Med. Imaging 12, 10. doi:10.1186/1471-2342-12-10. 22591335 PMC3368740

[R112] Niida R, Yamagata B, Niida A, Uechi A, Matsuda H, Mimura M, 2018. Aberrant anterior thalamic radiation structure in bipolar disorder: a diffusion tensor tractography study. Front. Psychiatry 9. doi:10.3389/fpsyt.2018.00522. PMC6207644 30405460

[R113] Nowacki A, Schlaier J, Debove I, Pollo C, 2018. Validation of diffusion tensor imaging tractography to visualize the dentato-rubro-thalamic tract for surgical planning. J. Neurosurg 130, 99–108. doi:10.3171/2017.9.JNS171321. 29570012

[R114] Ohoshi Y, Takahashi S, Yamada S, Ishida T, Tsuda K, Tsuji T, Terada M, Shinosaki K, Ukai S, 2019. Microstructural abnormalities in callosal fibers and their relationship with cognitive function in schizophrenia: a tract-specific analysis study. Brain Behav 9, e01357. doi:10.1002/brb3.1357. 31283112 PMC6710197

[R115] Panesar SS, Yeh F-C, Jacquesson T, Hula W, Fernandez-Miranda JC, 2018. A quantitative tractography study into the connectivity, segmentation and laterality of the human inferior longitudinal fasciculus. Front. Neuroanat 12. doi:10.3389/fnana.2018.00047. PMC5996125 29922132

[R116] Pascalau R, Popa Stănilă R, Sfrângeu S, Szabo B, 2018. Anatomy of the limbic white matter tracts as revealed by fiber dissection and tractography. World Neurosurg 113, e672–e689. doi:10.1016/j.wneu.2018.02.121. 29501514

[R117] Pascual-Diaz S, Varriano F, Pineda J, Prats-Galino A, 2020. Structural characterization of the extended frontal aslant tract trajectory: a ML-validated laterality study in 3T and 7T. Neuroimage 222, 117260. doi:10.1016/j.neuroimage.2020.117260. 32798677

[R118] Peltier J, Verclytte S, Delmaire C, Pruvo J-P, Havet E, Le Gars D, 2011. Microsurgical anatomy of the anterior commissure: correlations with diffusion tensor imaging fiber tracking and clinical relevance. Oper. Neurosurg 69, ons241–ons247. doi:10.1227/NEU.0b013e31821bc822. 21499149

[R119] Peng H, Cirstea CM, Kaufman CL, Frey SH, 2019a. Microstructural integrity of corticospinal and medial lemniscus tracts: insights from diffusion tensor tractography of right-hand amputees. J. Neurophysiol 122, 316–324. doi:10.1152/jn.00316.2018. 31116678 PMC6689782

[R120] Peng H, Cirstea CM, Kaufman CL, Frey SH, 2019b. Microstructural integrity of corticospinal and medial lemniscus tracts: insights from diffusion tensor tractography of right-hand amputees. J. Neurophysiol 122, 316–324. doi:10.1152/jn.00316.2018. 31116678 PMC6689782

[R121] Phillips O, Sanchez-Castaneda C, Elifani F, Maglione V, Di Pardo A, Caltagirone C, Squitieri F, Sabatini U, Di Paola M, 2013. Tractography of the corpus callosum in huntington’s disease. PLoS One 8. doi:10.1371/journal.pone.0073280. PMC3760905 24019913

[R122] Portegies JM, Fick RHJ, Sanguinetti GR, Meesters SPL, Girard G, Duits R, 2015. Improving fiber alignment in HARDI by combining contextual PDE flow with constrained spherical deconvolution. PLoS One 10. doi:10.1371/journal.pone.0138122, e0138122–e0138122. 26465600 PMC4605742

[R123] Radwan A, Nachtergaele P, Swinnen S, Decramer T, Uytterhoeven M, van Loon J, Theys T, Sunaert S, 2019. The temporo-insular projection system: a multisubject fiber tractography study using connectome diffusion da. In: Proceedings of the International Society of Magnetic Resonance in Medicine, ISMRM 27th Annual Meeting, Leiden, NL, p. 34. doi:10.13140/RG.2.2.20513.76642 34.

[R124] Re TJ, Levman J, Lim AR, Righini A, Grant PE, Takahashi E, 2017. High-angular resolution diffusion imaging tractography of cerebellar pathways from newborns to young adults. Brain Behav. 7, e00589. doi:10.1002/brb3.589. 28127511 PMC5256176

[R125] Rheault F, De Benedictis A, Daducci A, Maffei C, Tax CMW, Romascano D, Caverzasi E, Morency FC, Corrivetti F, Pestilli F, Girard G, Theaud G, Zemmoura I, Hau J, Glavin K, Jordan KM, Pomiecko K, Chamberland M, Barakovic M, Goyette N, Poulin P, Chenot Q, Panesar SS, Sarubbo S, Petit L, Descoteaux M, 2020. Tractostorm: the what, why, and how of tractography dissection reproducibility. Hum. Brain Mapp 41, 1859–1874. doi:10.1002/hbm.24917. 31925871 PMC7267902

[R126] Rheault F, St-Onge E, Sidhu J, Maier-Hein K, Tzourio-Mazoyer N, Petit L, Descoteaux M, 2019. Bundle-specific tractography with incorporated anatomical and orientational priors. Neuroimage 186, 382–398. doi:10.1016/j.neuroimage.2018.11.018. 30453031

[R127] FreeSurferWiki, 2020. FreeSurferMethodsCitation - Free Surfer Wiki [WWW Document] URL https://surfer.nmr.mgh.harvard.edu/fswiki/FreeSurferMethodsCitation

[R128] Rorden C, Hanayik T, 2022. Surf Ice

[R129] Salamon N, Sicotte N, Drain A, Frew A, Alger JR, Jen J, Perlman S, Salamon G, 2007. White matter fiber tractography and color mapping of the normal human cerebellum with diffusion tensor imaging. J. Neuroradiol 34, 115–128. doi:10.1016/j.neurad.2007.03.002. 17481730

[R130] Schilling KG, Rheault F, Petit L, Hansen CB, Nath V, Yeh FC, Girard G, Barakovic M, Rafael-Patino J, Yu T, Fischi-Gomez E, Pizzolato M, Ocampo-Pineda M, Schiavi S, Canales-Rodríguez EJ, Daducci A, Granziera C, Innocenti G, Thiran JP, Mancini L, Wastling S, Cocozza S, Petracca M, Pontillo G, Mancini M, Vos SB, Vakharia VN, Duncan JS, Melero H, Manzanedo L, Sanz-Morales E, Peña-Melián Á, Calamante F, Attyé A, Cabeen RP, Korobova L, Toga AW, Vijayakumari AA, Parker D, Verma R, Radwan A, Sunaert S, Emsell L, Luca AD, Leemans A, Bajada CJ, Haroon H, Azadbakht H, Chamberland M, Genc S, Tax CMW, Yeh PH, Srikanchana R, Mcknight C, Yang JYM, Chen J, Kelly CE, Yeh CH, Cochereau J, Maller JJ, Welton T, Almairac F, Seunarine KK, Clark CA, Zhang F, Makris N, Golby A, Rathi Y, O’Donnell LJ, Xia Y, Aydogan DB, Shi Y, Fernandes FG, Raemaekers M, Warrington S, Michielse S, Ramírez-Manzanares A, Concha L, Aranda R, Meraz MR, Lerma-Usabiaga G, Roitman L, Fekonja LS, Calarco N, Joseph M, Nakua H, Voineskos AN, Karan P, Grenier G, Legarreta JH, Adluru N, Nair VA, Prabhakaran V, Alexander AL, Kamagata K, Saito Y, Uchida W, Andica C, Masahiro A, Bayrak RG, Wheeler-Kingshott CAMG, D’Angelo E, Palesi F, Savini G, Rolandi N, Guevara P, Houenou J, López-López N, Mangin JF, Poupon C, Román C, Vázquez A, Maffei C, Arantes M, Andrade JP, Silva SM, Raja R, Calhoun VD, Caverzasi E, Sacco S, Lauricella M, Pestilli F, Bullock D, Zhan Y, Brignoni-Perez E, Lebel C, Reynolds JE, Nestrasil I, Labounek R, Lenglet C, Paulson A, Aulicka S, Heilbronner S, Heuer K, Anderson AW, Landman BA, Descoteaux M, 2021b. Tractography dissection variability: what happens when 42 groups dissect 14 white matter bundles on the same dataset? NeuroImage 243. doi:10.1101/2020.10.07.321083, 118502. 34433094 PMC8855321

[R131] Schilling KG, Tax CM, Rheault F, Hansen CB, Yang Q, Yeh FC, Cai LY, Anderson AW, Landman BA, 2021a. Fiber tractography bundle segmentation depends on scanner effects, vendor effects, acquisition resolution, diffusion sampling scheme, diffusion sensitization, and bundle segmentation workflow. NeuroImage 242 (17). doi:10.1101/2021.03.17.435872, 435872. PMC9933001 34358660

[R132] Schurr R, Filo S, Mezer AA, 2019. Tractography delineation of the vertical occipital fasciculus using quantitative T1 mapping. Neuroimage 202, 116121. doi:10.1016/j.neuroimage.2019.116121, 116121. 31472252

[R133] Scilpy documentation [WWW Document], 2021. URL https://scilpy.readthedocs.io/en/latest/ (accessed 2.15.21).

[R134] Seltzer B, Pandya DN, 1984. Further observations on parieto-temporal connections in the rhesus monkey. Exp. Brain Res 55, 301–312. doi:10.1007/BF00237280. 6745368

[R135] Skare S, Bammer R, 2010. Jacobian weighting of distortion corrected EPI data, in: Proceedings of the 18th Annual Meeting of International Society for Magnetic Resonance in Medicine Stockholm, Sweden, p. 5603.

[R136] Smith RE, Tournier J-D, Calamante F, Connelly A, 2015. SIFT2: enabling dense quantitative assessment of brain white matter connectivity using streamlines tractography. Neuroimage 119, 338–351. doi:10.1016/j.neuroimage.2015.06.092. 26163802

[R137] Smith RE, Tournier JD, Calamante F, Connelly A, 2013. SIFT: spherical-deconvolution informed filtering of tractograms. Neuroimage 67, 298–312. doi:10.1016/j.neuroimage.2012.11.049. 23238430

[R138] Smith RE, Tournier JD, Calamante F, Connelly A, 2012. Anatomically-constrained tractography: Improved diffusion MRI streamlines tractography through effective use of anatomical information. Neuroimage 62, 1924–1938. doi:10.1016/J.NEUROIMAGE.2012.06.005. 22705374

[R139] Smith SM, Jenkinson M, Woolrich MW, Beckmann CF, Behrens TEJ, Johansenberg H, Bannister PR, Luca MD, Drobnjak I, Flitney DE, Niazy RK, Saunders J, Vickers J, Zhang Y, Stefano ND, Brady JM, Matthews PM, 2004. Advances in functional and structural MR image analysis and implementation as. FSL 23, 208–219. doi:10.1016/j.neuroimage.2004.07.051. 15501092

[R140] Soares JM, Marques P, Alves V, Sousa N, 2013. A hitchhiker’s guide to diffusion tensor imaging. Front. Neurosci 7, 1–14. doi:10.3389/fnins.2013.00031. 23486659 PMC3594764

[R141] Stieltjes B, Kaufmann WE, van Zijl PCM, Fredericksen K, Pearlson GD, Solaiyappan M, Mori S, 2001. Diffusion tensor imaging and axonal tracking in the human brainstem. Neuroimage 14, 723–735. doi:10.1006/nimg.2001.0861. 11506544

[R142] Strange BA, Witter MP, Lein ES, Moser EI, 2014. Functional organization of the hippocampal longitudinal axis. Nat. Rev. Neurosci 15, 655–669. doi:10.1038/nrn3785. 25234264

[R143] Thiebaut de Schotten M, ffytche DH, Bizzi A, Dell’Acqua F, Allin M, Walshe M, Murray R, Williams SC, Murphy DGM, Catani M, 2011. Atlasing location, asymmetry and inter-subject variability of white matter tracts in the human brain with MR diffusion tractography. Neuroimage 54, 49–59. doi:10.1016/j.neuroimage.2010.07.055. 20682348

[R144] Toselli B, Tortora D, Severino M, Arnulfo G, Canessa A, Morana G, Rossi A, Fato MM, 2017. Improvement in white matter tract reconstruction with constrained spherical deconvolution and track density mapping in low angular resolution data: a pediatric study and literature review. Front. Pediatr 5, 182. doi:10.3389/fped.2017.00182. 28913326 PMC5582070

[R145] Tourbier S, Aleman-Gomez Y, Griffa A, Bach Cuadra M, Hagmann P, 2019. sebastientourbier/multiscalebrainparcellator: multi-scale brain parcellator v1.1.1 Zenodo doi:10.5281/zenodo.3627097.

[R146] Tournier JD, Calamante F, Connelly A, 2010. Improved probabilistic streamlines tractography by 2nd order integration over fibre orientation distributions. In: Proceedings of the International Society for Magnetic Resonance in Medicine ISMRM, 18.

[R147] Tournier JD, Mori S, Leemans A, Morgan RH, 2011. Imaging methodology-review diffusion tensor imaging and beyond. Magn. Reson. Med 65, 1532–1556. doi:10.1002/mrm.22924. 21469191 PMC3366862

[R148] Tournier JD, Smith RE, Raffelt DA, Tabbara R, Dhollander T, Pietsch M, Christiaens D, Jeurissen B, Yeh CH, Connelly A, 2019. MRtrix3: a fast, flexible and open software framework for medical image processing and visualisation. Neuroimage 202. doi:10.1016/j.neuroimage.2019.116137, 116137–116137. 31473352

[R149] Tsao H, Pannek K, Boyd RN, Rose SE, 2015. Changes in the integrity of thalamocortical connections are associated with sensorimotor deficits in children with congenital hemiplegia. Brain Struct. Funct 220, 307–318. doi:10.1007/s00429-013-0656-x. 24146132

[R150] van Baarsen KM, Kleinnijenhuis M, Jbabdi S, Sotiropoulos SN, Grotenhuis JA, van Cappellen van Walsum AM, 2016. A probabilistic atlas of the cerebellar white matter. Neuroimage 124, 724–732. doi:10.1016/j.neuroimage.2015.09.014. 26385011

[R151] Van Essen DC, Ugurbil K, Auerbach E, Barch D, Behrens TEJ, Bucholz R, Chang A, Chen L, Corbetta M, Curtiss SW, Della Penna S, Feinberg D, Glasser MF, Harel N, Heath AC, Larson-Prior L, Marcus D, Michalareas G, Moeller S, Oostenveld R, Petersen SE, Prior F, Schlaggar BL, Smith SM, Snyder AZ, Xu J, Yacoub E, 2012. The human connectome project: a data acquisition perspective. Neuroimage 62, 2222–2231. doi:10.1016/j.neuroimage.2012.02.018. 22366334 PMC3606888

[R152] van Meer N, Houtman AC, Van Schuerbeek P, Vanderhasselt T, Milleret C, ten Tusscher MP, 2016. Interhemispheric connections between the primary visual cortical areas via the anterior commissure in human callosal agenesis. Front. Syst. Neurosci 10. doi:10.3389/fnsys.2016.00101. PMC5183601 28082873

[R153] Veraart J, Fieremans E, Novikov DS, 2016. Diffusion MRI noise mapping using random matrix theory. Magn. Reson. Med 76, 1582–1593. doi:10.1002/mrm.26059. 26599599 PMC4879661

[R154] Verhoeven JS, Sage CA, Leemans A, Van Hecke W, Callaert D, Peeters R, De Cock P, Lagae L, Sunaert S, 2010. Construction of a stereotaxic DTI atlas with full diffusion tensor information for studying white matter maturation from childhood to adolescence using tractography-based segmentations. Hum. Brain Mapp 31, 470–486. doi:10.1002/hbm.20880. 19957267 PMC6870577

[R155] Voogd J, Paxinos G, Mai JK, 2004. CHAPTER 11 - cerebellum and precerebellar nuclei. In: The Human Nervous System Academic Press, San Diego, pp. 321–392. doi:10.1016/B978-012547626-3/50012-0.

[R156] Wakana S, Caprihan A, Panzenboeck MM, Fallon JH, Perry M, Gollub RL, Hua K, Zhang J, Jiang H, Dubey P, Blitz A, van Zijl P, Mori S, 2007. Reproducibility of Quantitative Tractography Methods Applied to Cerebral White Matter. NeuroImage 36, 630–644. doi:10.1016/j.neuroimage.2007.02.049. 17481925 PMC2350213

[R157] Wakana S, Jiang H, Nagae-Poetscher LM, Zijl PCM van, Mori S, 2004. Fiber tract–based atlas of human white matter anatomy. Radiology doi:10.1148/radiol.2301021640. 14645885

[R158] Wang X, Pathak S, Stefaneanu L, Yeh FC, Li S, Fernandez-Miranda JC, 2016. Subcomponents and connectivity of the superior longitudinal fasciculus in the human brain. Brain Struct. Funct 221, 2075–2092. doi:10.1007/s00429-015-1028-5. 25782434

[R159] Wang Y, Fernández-Miranda JC, Verstynen T, Pathak S, Schneider W, Yeh FC, 2013. Rethinking the role of the middle longitudinal fascicle in language and auditory pathways. Cereb. Cortex 23, 2347–2356. doi:10.1093/cercor/bhs225. 22875865

[R160] Warrington S, Bryant KL, Khrapitchev AA, Sallet J, Charquero-Ballester M, Douaud G, Jbabdi S, Mars RB, Sotiropoulos SN, 2020. XTRACT - standardised protocols for automated tractography in the human and macaque brain. Neuroimage 217, 116923. doi:10.1016/j.neuroimage.2020.116923. 32407993 PMC7260058

[R161] Wasserthal J, Neher P, Maier-Hein KH, 2018. TractSeg - fast and accurate white matter tract segmentation. Neuroimage 183, 239–253. 30086412 10.1016/j.neuroimage.2018.07.070

[R162] Wilde EA, Bigler ED, Haider JM, Chu Z, Levin HS, Li X, Hunter JV, 2006. Vulnerability of the anterior commissure in moderate to severe pediatric traumatic brain injury. J. Child Neurol 21, 769–776. doi:10.1177/08830738060210090201. 16970884

[R163] Wilkins B, Lee N, Gajawelli N, Law M, Leporé N, 2015. Fiber estimation and tractography in diffusion MRI: Development of simulated brain images and comparison of multi-fiber analysis methods at clinical b-values. Neuroimage 109, 341–356. doi:10.1016/j.neuroimage.2014.12.060. 25555998 PMC4600612

[R164] Wu W, Rigolo L, O’Donnell LJ, Norton I, Shriver S, Golby AJ, 2012. Visual pathway study using in vivo diffusion tensor imaging tractography to complement classic anatomy. Oper. Neurosurg 70, ons145–ons156. doi:10.1227/NEU.0b013e31822efcae. PMC3236807 21808220

[R165] Wu Y, Sun D, Wang Yong, Wang Yibao, 2016a. Subcomponents and connectivity of the inferior fronto-occipital fasciculus revealed by diffusion spectrum imaging fiber tracking. Front. Neuroanat 10. doi:10.3389/fnana.2016.00088. PMC5033953 27721745

[R166] Wu Y, Sun D, Wang Y, Wang Y, Ou S, 2016b. Segmentation of the cingulum bundle in the human brain: a new perspective based on DSI tractography and fiber dissection study. Front. Neuroanat 10. doi:10.3389/fnana.2016.00084. PMC5013069 27656132

[R167] Xiao Y, Beriault S, Pike GB, Collins DL, 2012. Multicontrast multiecho FLASH MRI for targeting the subthalamic nucleus. Magn. Reson. Imaging 30, 627–640. doi:10.1016/j.mri.2012.02.006. 22503090

[R168] Xiao Y, Fonov V, Bériault S, Al Subaie F, Chakravarty MM, Sadikot AF, Pike GB, Collins DL, 2015. Multi-contrast unbiased MRI atlas of a Parkinson’s disease population. Int. J. Comput. Assist. Radiol. Surg 10, 329–341. doi:10.1007/s11548-014-1068-y. 24841147

[R169] Xiao Y, Fonov V, Chakravarty MM, Beriault S, Al Subaie F, Sadikot A, Pike GB, Bertrand G, Collins DL, 2017. A dataset of multi-contrast population-averaged brain MRI atlases of a Parkinson׳s disease cohort. Data Brief 12, 370–379. doi:10.1016/j.dib.2017.04.013. 28491942 PMC5413210

[R170] Yeatman JD, Weiner KS, Pestilli F, Rokem A, Mezer A, Wandell BA, 2014. The vertical occipital fasciculus: a century of controversy resolved by in vivo measurements. Proc. Natl. Acad. Sci 111, E5214–E5223. 25404310 10.1073/pnas.1418503111 PMC4260539

[R171] Yeh FC, Panesar S, Fernandes D, Meola A, Yoshino M, Fernandez-Miranda JC, Vettel JM, Verstynen T, 2018. Population-averaged atlas of the macroscale human structural connectome and its network topology. Neuroimage 178, 57–68. doi:10.1016/j.neuroimage.2018.05.027. 29758339 PMC6921501

[R172] Yendiki A, Panneck P, Srinivasan P, Stevens A, Zöllei L, Augustinack J, Wang R, Salat D, Ehrlich S, Behrens T, Jbabdi S, Gollub R, Fischl B, 2011. Automated probabilistic reconstruction of white-matter pathways in health and disease using an atlas of the underlying anatomy. Front. Neuroinform 5. doi:10.3389/fninf.2011.00023. PMC3193073 22016733

[R173] Younes K, Hasan KM, Kamali A, McGough CE, Keser Z, Hasan O, Melicher T, Kramer LA, Schulz PE, 2019. Diffusion tensor imaging of the superior thalamic radiation and cerebrospinal fluid distribution in idiopathic normal pressure hydrocephalus. J. Neuroimaging 29, 242–251. doi:10.1111/jon.12581. 30461106

[R174] Zhang F, Wu Y, Norton I, Rathi Y, Golby AJ, O’Donnell LJ, 2019. Test–retest reproducibility of white matter parcellation using diffusion MRI tractography fiber clustering. Hum. Brain Mapp 40, 3041–3057. doi:10.1002/hbm.24579. 30875144 PMC6548665

[R175] Zhang Y, Zhang J, Oishi K, Faria AV, Jiang H, Li X, Akhter K, Rosa-Neto P, Pike GB, Evans A, Toga AW, Woods R, Mazziotta JC, Miller MI, van Zijl PCM, Mori S, 2010. Atlas-guided tract reconstruction for automated and comprehensive examination of the white matter anatomy. Neuroimage 52, 1289–1301. doi:10.1016/j.neuroimage.2010.05.049. 20570617 PMC2910162

[R176] Zheng Y, Wang D, Ye Q, Zou F, Li Y, Kwok SC, 2020. Diffusion property and functional connectivity of superior longitudinal fasciculus underpin human metacognition. bioRxiv 03 (17), 994574. doi:10.1101/2020.03.17.994574. 33812946

